# Ukrainian–Russian bilingualism in the war-affected migrant and refugee communities in Austria and Germany: a survey-based study on language attitudes

**DOI:** 10.3389/fpsyg.2024.1364112

**Published:** 2024-05-23

**Authors:** Vladislava Warditz, Natalia Meir

**Affiliations:** ^1^Department of Slavic Studies, University of Potsdam, Potsdam, Germany; ^2^Department of English Literature and Linguistics, Bar-Ilan University, Ramat Gan, Israel

**Keywords:** bilingualism, languages and war, migrant post-colonial bilingualism, Ukrainian–Russian bilingualism, languages in diasporic communities, migration, refugees, language attitudes

## Abstract

**Introduction:**

This paper provides an initial exploration of Ukrainian–Russian bilingualism in the context of the war-affected migration from Ukraine to Austria and Germany. While extensive research exists on various aspects of Ukrainian– Russian bilingualism in relation to Ukraine itself, thus far no studies have been conducted on this bilingualism in the diasporic context, i.e., as a language of the first and subsequential generations with a migrant background in Austria and Germany.

**Methods:**

To address this research gap, our paper examines the language attitudes of two respondent groups with a Ukrainian background in the two countries: migrants and refugees who left Ukraine after 2014 and those who left after Russia’s invasion in February 2022. In the framework of a sociolinguistic survey, we describe their current attitudes regarding the use of Ukrainian and Russian, among others, in relation to the actual and intended use of the language(s) in the multilingual context of migration. The survey eliciting information on demographic information, language proficiency, language attitudes and language use was conducted on 406 Ukrainians in two host countries (Austria: *n* = 103; Germany: *n* = 306). First, we compared self-rated proficiency in Ukrainian and Russian as well as attitudes and use of these languages. Second, we applied a network modelling analysis to determine the nature of relationships between these variables.

**Results and discussion:**

The results indicated that proficiency in Ukrainian and in Russian were the strongest nodes in the model affecting language use and language attitudes toward the respective languages. Our data analysis focused on the pragmatic and symbolic value of Russian and Ukrainian playing a crucial role in the language vitality in multilingual settings. The paper discusses the imbalanced correlation of the symbolic and pragmatic value of Ukrainian and Russian in the diasporic Ukrainian communities. While Ukrainian has gained a higher symbolic status, Russian maintains a better pragmatic one, despite its negative symbolic status. However, we anticipate that the increasing symbolic value of Ukrainian and the diminishing value of Russian will lead to an increase in the use of Ukrainian also in Russian-dominant bilingual groups of Ukrainian migrants and refugees, even as an insider-code in hermetic minority groups.

## 1 Introduction

War affects the usage and status of languages. War not only challenges language use in multiethnic empires, multinational coalitions or refugee crises (translation, interpreting, standardization, etc.) (Zhdanova, 2009; Declercq and Walker, 2016; Walker and Declercq, 2016; Scheer, 2022), but since the 19th century, and especially in the 20th century, language has become the prime marker of national identity and the engine behind the creation of nation-states (e.g., Italy, Germany, the new independent states after WWI, the demise of the Soviet Union in 1991 and the Yugoslav Wars in the 1990s) (Miller, 2006; Leerssen, 2010; Connelly, 2020). Given the direct correlation between languages and nation-states, war between nation-states necessarily affects the relationship between languages and the cultural identity they represent, and is even invoked as a justification for war.

In this paper, we explore Ukrainian–Russian bilingualism within the context of the current war-affected migration from Ukraine to Austria and Germany, focusing on the language attitudes of the migrants and refugees who left Ukraine after 2014 and of those who left after Russia’s invasion in February 2022, respectively. In the framework of a sociolinguistic survey, we aim to document respondents’ attitudes regarding the use of Ukrainian and Russian in the multilingual context of migration. Thereby, we define language attitudes as evaluative reactions to language (Albarracin and Shavitt, 2018; Dragojevic et al., 2021), shedding light on essential factors affecting language maintenance in multilingual contexts (Bradley, 2013).

Based on the corpus of documented attitudes, we investigate whether (and if so, to what extent) the use and maintenance of Ukrainian and/or Russian in refugee and migrant communities are motivated by the pragmatic and/or symbolic value of languages and whether this has (potentially) changed due to the Russian–Ukrainian war. On the one hand, language use and language maintenance, especially of minority languages, can be driven by the symbolic value of this language rather than pragmatic ones. The symbolic value refers to “extrarational” or “emotional” associations evoked by a certain language, whereas the pragmatic value refers to its communicative value and comprises, therefore, its “rational,” “instrumental,” or “functional” dimensions (De Kadt, 1993; Rahman, 2001). A multilingual person might choose to use a particular language out of his/her desire to be associated with this language, as this language is a symbol of ethnic identification and cultural heritage, cf. e.g., the case of Circassian in Jordan (Abd El Jawad, 2006) or the case of English and Pinyin (Shang, 2020). On the other hand, the pragmatic value of language can also crucially affect language maintenance in the multilingual situation, especially by the contact of languages with different communicative range, i.e., of a pragmatically stronger and a pragmatically weaker language (De Kadt, 1993). This is often a case in post-colonial settings with, e.g., English or Russian as a former dominant societal language and a *lingua franca* in the given context of multilingualism and various minority languages, cf. for the post-Soviet space see the most recently edited volume (Forker and Grenoble, 2021) or classical encyclopedic works such as (Neroznak, 1994, 1995). Whereas pragmatic and symbolic considerations of language maintenance have received considerable attention in post-colonial settings, they have not been discussed in the context of war-affected migrant multilingualism. War-effected multilingualism as such has been addressed in studies focusing on language education in mono- or multilingual settings, especially in post-war eras or on the military use of languages during the war (for overview, cf. Walker and Declercq, 2016). These studies primarily look at the subject from a historical point of view, and do not build on a linguistic theoretical framework. Our study tries to fill this gap. As the first of its kind, our study wants to apply the post-colonial linguistic frame to migrant communities in times of war, and in so doing to inscribe this linguistic approach in the broader field of languages and war. With our exploratory study on post-Soviet, Russian-based bilingualism in migrant communities, we want to open the debate on war-affected migrant bilingualism as such. In particular, our study wants to contribute to the development of a (highly relevant) framework of *post*-*colonial bilingualism transferred to diasporas*, notably in war-affected diasporas where both home languages are used.

Thus, our study documents the dynamics of language attitudes in the war-affected diasporic communities and, at the same time, contributes to the theory formation of language maintenance in imbalanced multilingual settings.

Our paper is structured as follows: in the first section, on the basis of previous research, we describe the political and sociolinguistic dimensions of Ukrainian–Russian bilingualism in Ukraine in the context of the war. In the second section, we examine the demographic and linguistic dimensions of the Ukrainian diaspora in Austria and Germany, relevant for the language maintenance and vitality. The third section includes our research questions and hypotheses within the drawn theoretical framework, focusing on the discussion of the role of pragmatic and symbolic values of languages in the situation of (un) balanced multilingualism. The fourth section presents our empirical study (survey), and the fifth section discusses the main outcomes of our investigation summarized in the conclusion of the paper. The surveys in both languages (Russian and Ukrainian) are attached in Appendix.

## 2 Ukrainian–Russian bilingualism: sociolinguistic and political dimensions

In the well-established context of migrant language research, Ukrainian in general and in Austria and Germany in particular has hardly been studied. However, Ukrainian as a language of rapidly expanding war-affected migrant and refugee communities in both countries presents a special case of (Slavic) migrant languages. Ukrainian, the second largest Slavic language (cf. 45 million speakers), has a long history of imbalanced functional distribution with other (dominant) languages, such as Polish, German and Russian. Taking into account the complexity of Ukraine’s multilinguistic history and the establishment of the Ukrainian language within this framework (Shevelov, 1966; Miller, 2006), we focus on the current issues of Ukrainian–Russian bilingualism. For over 350 years, Ukrainian has co-existed with Russian as the dominant language, first in Muscovy and the Russian Empire, and after 1922 (respectively 1939 for the Western parts of the country) in the USSR. During the Soviet period, and especially after 1933, in major communicative domains such as administration, education, science, culture and the army, Ukrainian played either a secondary role or was completely suppressed. The resulting imbalanced Ukrainian–Russian bilingualism, to the detriment of Ukrainian, and, as a consequence, the emergence of a mixed Ukrainian–Russian code, known as *suržyk* (Trub, 2000: 47–49) are therefore characteristic for the language situation in Ukraine (Tkachenko, 1999; Taranenko, 2001; Burda, 2002; see also Bilaniuk, 2005; Besters Dilger, 2009; Kulyk, 2017; Sokolova and Zalizniak, 2018; Zhabotynska, 2018; Masenko, 2020a). In our paper, we use the terms “Russian-speaking” and “Ukrainian-speaking” to indicate the dominant language in the bilinguals under scrutiny. It does not refer to their ethnic identity or political sympathies.

After Ukraine’s independence in 1991 and the establishment of Ukrainian as the sole official language, Ukrainian–Russian bilingualism has retained its relevance not only in language policy, but also in political debates (Olszański, 2012: 41–49; Moser, 2013a; Shevchenko, 2014; cf. the discussion of Language Laws of Ukraine in Moser, 2015; Csernicskó and Fedinec, 2016; Csernicskó and Kontra, 2022). If the assumption of a language conflict in Ukraine has become almost commonplace, this is due, on the one hand, to the permanent allegations by the Russian government about the discrimination, if not persecution, of Russians or Russian speakers in the country. On the other hand, it stems from the continuous controversies in Ukraine itself over the status of Russian next to Ukrainian (Hentschel and Zeller, 2016: 636–637). At the same time, among the population, the language issue seemed to be much less conflictual, i.e., the political and ideological instrumentalization of languages had not (yet) influenced speakers’ attitudes, cf. the analysis of the pre-crisis survey data (Hentschel and Brüggemann, 2015) and of the survey data after 2014 (Hentschel and Zeller, 2016). According to Marusyk, the uniqueness of the language situation in Ukraine lies in the split between official and popular language policies (“державна і громадська мовні політики”) which often do not complement but oppose each other (Marusyk, 2019: 175; cf. the papers discussing Ukrainian-related issues of language policies within a cross-linguistic framework in Azhniuk, 2019).

However, a recent sociological study (Kulyk, 2023) shows that the use of languages by the Ukrainian population is currently undergoing profound changes. These changes obviously have been going on for some time, at the least since 2014, cf. analysis of Ukrainian legislative acts and media events regarding language policies of the state in Marusyk (2019), or results of a sociolinguistic survey in Sokolova (2019): 173–174. Sokolova documented a significant increase (by over 15%) in the number of respondents who saw Ukrainian as the only state language, although more than a quarter of Ukrainian citizens still wanted Russian to have the status of at least a second official language. According to Kulyk (2023), even in the predominantly Russian-speaking East and South of the country,^1^ i.e., in the regions with Russian-dominant bilingualism, many people have reacted to Russia’s large-scale invasion of 2022 by switching to Ukrainian in private and/or public conversations. In this way, these regions are becoming more similar to the Center and the West, creating greater unity and testifying to the resilience of the Ukrainian nation. Kulyk (2023): 5, however, could not determine the extent of this language change, as he had doubts about how honestly the survey was answered. He assumed that some people were unintentionally stating their wishful thinking rather than describing the real situation and that some were deliberately distorting habits that had become “politically incorrect” in the context of the war. As Kulyk tried to overcome these quite predictable limitations of self-reported data through formulating the questions in a special way, we understand his observations as reliable enough for the further studies.

While this linguistic issue has been researched in relation to Ukraine itself, it has not been studied in relation to the Ukrainian diasporic communities across the world. The present paper aims to fill this gap by examining language attitudes towards Russian and Ukrainian in the context of the current war-affected migration from Ukraine to Austria and Germany. Hereby, we consider refugees and migrants with a Ukrainian background, i.e., (former) Ukrainian citizens regardless of which language they speak or proclaim as being their L1.

Ukraine has always been a multilingual country, with currently 13 officially renown minority languages, among others, Hungarian, Romanian, Bulgarian, Russian, Polish, Yiddish, and Gagauzian. Russian has been the largest minority language, often used also as a *lingua franca*, especially in urban settings (Kulyk, 2017; Masenko, 2020b). Thereby, a distribution of both languages, Ukrainian and Russian, already in the pre-war country was more complex than simply dichotomic. The East and South of Ukraine are predominantly Russian-speaking and the Center and West predominantly Ukrainian-speaking, however, smaller parts of population, to a largest extent in the northeastern regions, use Ukrainian–Russian mixed speech called *suržyk* in everyday conversation (for overview, see Del Gaudio, 2015). Thus, there are rather gradual transitions between the three codes used in Ukraine: Ukrainian, Russian and *suržyk*. Nevertheless, while all three codes have a significant linguistic dimension, only Ukrainian and Russian have a political dimension. In the context of the war, Ukrainian as the state language (“deržavna mova”) of the independent Ukraine, on the one hand, and Russian as a language of the military adversary’s country, on the other hand, have assumed a (respectively positive and negative) symbolic status (Kulyk, 2023).

Within the given linguistic and political dimensions, language attitudes of the Ukrainian diaspora remain unstudied, although Ukrainian diasporic communities play a significant role in the maintenance of Ukrainian (cf. Moser, 2013b). While speakers’ attitudes towards Ukrainian and Russian seem to be radicalized in the homeland accordingly to the symbolic value of both languages (Kulyk, 2023), it can be assumed that language attitudes of the Ukrainian diaspora have also undergone changes.

Based on the survey data, the present study examines language attitudes to Ukrainian and Russian within the framework of Ukrainian–Russian bilingualism in the Ukrainian war-affected diasporic communities in Austria and Germany. The study focuses on the (potential) problems vs. conflicts of the postcolonial Ukrainian–Russian bilingualism in Ukraine transferred in the Ukrainian diaspora and, accordingly, in the new context of multilingualism, namely with German as a societal language, on the one hand, and Ukrainian vs. Russian as a migrant heritage language, on the other hand. As pointed out above, we investigate Ukrainian–Russian bilingualism through the prism of post-colonial studies that rarely include multifaceted post-Soviet cases (Stolz, 2015) and, therefore, contributes to their involvement in the framework of colonial linguistics.

### 2.1 The Ukrainian diaspora in Austria and Germany: demography and language vitality

Originally defined as “that which makes a group likely to behave as a distinctive and active collective entity in intergroup situations” (Giles et al., 1977: 308), *linguistic vitality* encompasses three structural factors: demographic representation, institutional support, and the prestige of the language spoken by a community.^2^ The concept, initially developed within the research field of (often endangered) indigenous and colonial languages (cf. Bourhis et al., 2019), has also been applied to describe the linguistic vitality of the migrant heritage languages (Achterberg, 2005; Brown and Sachdev, 2009). In this section, we will address these three factors of vitality, whereby the chosen focus on the (im) balance between symbolic and pragmatic values of languages is supposed to be especially relevant in the context of war.

The war in Ukraine has created a new geopolitical situation which directly impacts the political, social and linguistic situation in Europe, particularly in Austria and Germany. Although the Polish and Russian diaspora remain the largest Slavic migrant communities in Germany, and the Serbian diaspora in Austria, the influx of migrants and refugees from Ukraine has been growing since 2014 (the annexation of Crimea by Russia) and has become massive since 2022. Since the beginning of the invasion on February 24, 2022, Germany has provided shelter to some 1,114,070 Ukrainians and Austria to 68,700 Ukrainians (Statista, 2023), October 29th, and it is expected that hundreds of thousands more will follow. In Austria, Ukrainian citizens accounted for the strongest growth of all foreign citizens (+66,899 people) in 2022. Accordingly, at the beginning of 2023, a total of 79,572 Ukrainian citizens were the ninth-largest migrant community in the country (*ibid.*). In comparison, in Germany, a total of 135,000 Ukrainian citizens were living at the end of 2020 (Destatis, 2022), March, 1, so the growth of the Ukrainian diaspora in the country is also significant. As a result, the Ukrainian presence in Austria and Germany has been rapidly increasing.

The exact number of refugees from Ukraine who have reached or left Germany or Austria cannot be determined with certainty. Some people may have traveled on or returned to Ukraine. Although the quoted statistical data do not say anything about the vitality of Slavic languages in diasporic communities, empirical studies indicate that the Slavic diaspora including Ukrainian have a strong tendency to maintain their heritage languages (for Germany: Achterberg, 2005; for Canada: Halko Addley and Khanenko Friesen, 2019; for Israel: Meir et al., 2021). Achterberg’s (2005) empirical sociolinguistic study, to date the only study on vitality of Slavic heritage languages in Germany, shows a high vitality of Ukrainian in comparison with Russian, which is reported to be the most vital Slavic heritage language in Germany (Achterberg, 2005: 161). However, Ukrainian less often than Russian has a status of a first family language in migrant families with Ukrainian background (Achterberg, 2005: 163). Even if Ukrainian is clearly less used than Russian in a daily communication of Ukrainian vs. Russian heritage speakers (ca. ½ and ca. 1/3 respectively) in Germany, its vitality index consisting of such parameters as speakers’ competence, their attitudes, functionality etc. is partly higher than the vitality index of heritage Russian, especially with regard to the parameter of identity (Achterberg, 2005: 242). Given the current situation, it is plausible to anticipate an increase in the use of Ukrainian as a marker of national identity. A rapid increase in refugees from Ukraine to Germany is also expected (Albrecht and Panchenko, 2022). Consequently, the linguistic study of Ukrainian outside Ukraine gains social and political relevance.

Notwithstanding this, Ukrainian as a migrant and/or heritage language in Austria and Germany has not been studied before. The existing studies relate to the USA and Canada and describe Ukrainian in contact with English (e.g., Budzhak Jones, 1998 and more recently: Mykhaylyk and Ytterstad, 2017; Nagy, 2018; Friedman, 2023). In Austria and Germany, Russian and Polish, respectively Serbian and Croatian as languages of the largest Slavic migrant communities have been studied in depth (for an overview, see Warditz, 2020), but Ukrainian has not drawn any scholarly interest. There are no studies available on Ukrainian in Austria and Germany (or for that matter in the whole of Europe). In this paper, we aim to fill the gap focusing on the language attitudes of the two respondent groups with Ukrainian background in Austria and Germany.

For characteristics of the sociolinguistic framework of the migrant heritage languages, the following factors are crucial. As all migrant heritage languages, Ukrainian (and Russian as the largest minority language among Ukrainian migrants and refugees) interact in a new multilingual environment, in our case, in Austria and Germany first of all with German as a societal language. Due to the well-known functional distribution of languages in the context of migration, migrant heritage language is used to a different extent only in private communication, e.g., in the family, while the societal language is used in official and partly in unofficial settings. In this sociolinguistic framework, the scope of everyday use of migrant heritage languages will be reduced (cf. Achterberg, 2005 for Slavic languages in Germany). Furthermore, this language situation has led, in turn, to language changes on all levels of the language (phonetics/phonology, morphology, syntax, semantics, lexicon, pragmatics) (Thomason, 2008, 2017 following Thomason and Kaufman, 1988). Added to this is the lack of prestige of the migrant heritage languages used to a different extent only in unofficial diasporic communication. Therefore, the vitality of migrant heritage languages, especially up to the second generation of speakers is endangered to a variable extent.

As for other factors relevant to the vitality of language(s) in a multilingual environment, Ukrainian and Russian, as well as the languages of other diasporic communities, lack legal status in both in Austria and Germany. This means, in turn, that even as languages of demographically well-represented diasporic communities, they do not enjoy institutional support, in contrast to officially designated minority languages, such as Sorbian, Danish, Low German (Plattdeutsch) or Romani in Germany. In the absence of institutional support, language maintenance and transgenerational transmission of a heritage language are up to the commitment of the corresponding community. Hence, language attitudes toward the heritage language, its significance for the identity and eventually its pragmatical value play a key role for language vitality in the given sociolinguistic situation.

In the case of Ukrainian, predictability of its vitality in the current and subsequential generations of diaspora is more complicated than for other Slavic heritage languages. Unlike most of the others, Ukrainian has already existed in a longstanding contact with another language, Russian. Apart from that, most Ukrainians are bilingual, i.e., they speak both languages, albeit with varying proficiency (Kulyk, 2017), whereby it should be expected that they transfer their well-established Ukrainian–Russian bilingualism into the country of immigration where both languages can be used. Therefore, prediction of the vitality of Ukrainian in diasporic communities is related to the question of (un)balanced Ukrainian–Russian bilingualism. Accordingly, the concept of pragmatic and symbolic power vs. value of language(s) used in a multilingual situation is relevant for our study. The concept will be discussed in the next section.

## 3 Concepts, research questions and methods of the present study

This paper aims to document and evaluate the language attitudes of Ukrainian refugees and migrants in Austria and Germany in relation to their use of Ukrainian and Russian. We utilized a survey-based analysis to conduct an exploratory investigation. For the most recent overview of the research methods in language attitudes (see Kircher and Zipp, 2022); for a specific focus on direct methods (see Kircher, 2022).

For our initial investigation, we selected these two countries for the following reasons. First of all, both countries have traditionally well-represented Slavic diaspora, that first emerged after the collapse of the Russian (1917) and Habsburg (1918) monarchies, then after World War II and with the onset of the mass-migration from the (post) socialistic Slavic countries in the 1970s and 1990s (cf. overviews in Warditz, 2013, 2020). Secondly, the high vitality index for Ukrainian in Germany has already been demonstrated within the pre-crisis community (Achterberg, 2005). Based on the demographic data of the pre-crisis Ukrainian diaspora in Austria, mainly consisting of Ukrainian-speaking migrants from Western Ukraine, we can assume here a correspondingly high vitality of Ukrainian as well. Thirdly, both countries are currently hosting Ukrainian refugees, with Germany hosting the largest number of Ukrainian in the European Union. However, there are also differences that can impact the vitality of both languages in the selected countries. In contrast to Austria, Germany has supported and promoted state-sponsored mass immigration of Russian–German “late resettlers” and Russian–Jewish “quota refugees” from the former USSR, the majority of whom were Russian speakers. This contributed to the establishment of Russian as a primary communication tool in post-Soviet migrant communities and facilitated its transgenerational maintenance. In Austria, prior to the admission of war refugees from Ukraine, immigration from the former USSR was predominantly individual. Therefore, the hegemony of Russian in post-Soviet migrant communities in Austria can be expected to be significantly less pronounced. Overall, the choice of Austria and Germany for this investigation provides a comprehensive foundation for studying Ukrainian diaspora and refugee communities, taking into account historical, linguistic, and demographic factors, as well as the current dynamics of the refugee crisis. This approach enhances the generalizability of findings and facilitates the identification of commonalities and distinctions within Ukrainian communities across different national contexts.

As the paper examines current attitudes of Ukrainian migrants and refugees regarding the use of Ukrainian–Russian bilingualism, for the aims of our study, the concept of pragmatic and symbolic power vs. value of language(s) is relevant. Pragmatic power is based “on the communicative dimensions of language” (De Kadt, 1993: 160), which is operationalized as language use across different contexts. Symbolic power refers to the association of a language with attributes that have a value, positive or negative, in the mind of the perceiver (Rahman, 2001: 57), for instance, English is associated with modernity, knowledge, and education in Pakistan, while Punjabi is not (Rahman, 2001: 57).

In the context of the post-Soviet migration, Russian has a higher pragmatic value as a language of the largest multiethnic diaspora and serves as a *lingua franca* between different ethnic diaspora from the former USSR whether Russian is their first or second language (Warditz, 2013; Levkovych, 2015).^3^ According to the in-depth sociological study (Panagiotidis, 2020), post-Soviet immigrants are the largest immigrant group in present-day German society. However, this heterogeneous entity comprises not only two major ethno-administrative categories: Russian–German “late resettlers” and Russian–Jewish “quota refugees” (Panagiotidis, 2020), but also descendants from the former Soviet national republics and autonomies, with Russian as a first or as a second language in the first generation and not unfrequently, in the second, cf. the study on the Kazakh diaspora across the world (Zhakupova, 2014). In this context, in migrant studies, the post-Soviet Russian-speaking diaspora has been defined as transnational (Kosmarskaya, 2005). Moreover, German for refugees is often taught by Russian-speaking migrants; they also work as volunteers in refugee-welcome programs in Austria and Germany. For pragmatic reasons, Russian may be used more frequently than Ukrainian.^4^ At the same time, we can expect growing tensions and as a result of increasing maintenance of Ukrainian due to its symbolic value shared also by Russian-dominant Ukrainians, i.e., it foreseeable that Ukrainian will be preserved even in families and groups not originally fluent in Ukrainian. Thus, the current Ukrainian diaspora faces a paradoxical situation regarding Ukrainian–Russian bilingualism. In the context of the war-affected migration, it is anticipated that pragmatically driven use of Russian may increase, potentially at the expense of the Ukrainian. Conversely, it can be expected that the use of Ukrainian as a sign of identity and solidarity with the homeland will grow, not least due to the negative symbolic value of Russian.^5^

In the next section, these hypothetic statements will be verified through the examination of the survey-based language attitudes of the Ukrainian diasporic communities in Austria and Germany.

The present study has been planned as a first step in a large-scale research project on the vitality of Ukrainian in the Ukrainian diaspora across the world. Accordingly, this paper aims to elucidate the following research questions:

1. What are the current attitudes of the respondent groups toward the Ukrainian and Russian languages? To what extent do the attitudes of refugees and migrants differ or overlap? Are there any observable differences between the migrant and refugee communities in Austria in Germany?
2. Whether and if so, to what extent, are the granted symbolic and pragmatic statuses of Ukrainian vs. Russian associated with the (socio) linguistic background of the respondents?

By addressing these research questions, the paper can further contribute to the discussion about predictions related to the maintenance and vitality of Ukrainian in the diasporic communities. The present study is therefore relevant for the investigation of the sustainable vitality of the Ukrainian language in two aspects: within the framework of Ukrainian–Russian bilingualism and in war-affected multilingual settings, both of these overlapping frameworks are entirely applicable to our study.

## 4 Language attitudes of the Ukrainian migrants and refugees in Austria and Germany: a survey-based analysis

### 4.1 Questionnaire

A novel questionnaire was developed specifically for this study and consisted of three sections: demographic information, language proficiency, and language attitudes. The questionnaire was modelled after previous questionnaires that gathered information on language attitudes and language use (e.g., Hentschel and Zeller, 2016; Kulyk, 2023). The first section of the survey included questions about the sociolinguistic background of respondents: age, gender, education level, year of arrival in Germany or Austria, place of residence in Germany or Austria, previous place of residence in Ukraine. The second section explored the language biography of the participants, asking whether they came from a bilingual family, what languages they heard in their childhood on a regular basis, as well as questions about knowledge of Ukrainian, Russian, German and/or any other additional languages. The third section focused on language attitudes toward the Ukrainian and Russian languages and included the questions presented in Table 1. For both languages, there were 11 Likert-type statements that respondents were asked to rate on a 1–5 scale corresponding to 1—“Strongly disagree,” 2—“Do not agree,” 3—“Difficult to answer,” 4—“Agree,” 5—“Strongly agree.” The section featured questions about identity and emotional connection, language vitality of the Ukrainian and Russian languages and their use in Austria and Germany, as well as changes in attitudes due to the Russian–Ukrainian war.

**Table 1:** Questionnaire: language use, attitudes and identity.

Q1: There are few opportunities to support this language in the host country: (A) Ukrainian, (B) Russian
Q2: I use more of this language in the host country: (A) Ukrainian, (B) Russian
Q3: I prefer using this language: (A) Ukrainian, (B) Russian
Q4: I lack vocabulary when speaking this language: (A) Ukrainian, (B) Russian
Q5: I feel I can express my emotions in this language: (A) Ukrainian, (B) Russian
Q6: This language evokes positive emotions in me: (A) Ukrainian, (B) Russian
Q7: I do not feel like myself when I speak this language: (A) Ukrainian, (B) Russian
Q8: Knowing this language is an important part of my identity: (A) Ukrainian, (B) Russian
Q9: It is important for me that my children know this language: (A) Ukrainian, (B) Russian
Q10: The war and the political events of recent years have changed my attitude toward the language for the worse: (A) Ukrainian, (B) Russian
Q11: I would like to stop using this language altogether: (A) Ukrainian, (B) Russian

The questionnaire was created in Google Forms. It was distributed on social networks, Facebook and Telegram and was available in Ukrainian and Russian. We collected survey responses in Austria and Germany in November–December 2023. The complete questionnaires in Ukrainian and Russian are available in Appendix.

### 4.2 Respondents: socio-demographic information

A total of 406 Ukrainians in the two countries (Austria: *n* = 103; Germany: *n* = 306) participated in the survey. We divided the participants further into two subgroups according to their migrant status. The first subgroup, labelled “Refugees,” consisted of Ukrainian refugees who arrived after February 24, 2022, i.e., after the beginning of Russian Federation’s invasion of Ukraine. The second subgroup, labelled “Migrants” was made up of Ukrainians who have been living in Germany or Austria for more than 1 year, i.e., those respondents who arrived in Austria and Germany before 2022.

During data pre-processing, we excluded data from 8 participants (2% of the entire data) due to incompleteness (e.g., missing current/previous place of residence) and age below 18 years (*n* = 3). To ensure sample homogeneity, we further excluded 6 participants, comprising 1.5% of the entire sample, who reported that their languages were other than Russian and Ukrainian (e.g., other: *n* = 2; Spanish & Russian: *n* = 1; Russian & German: *n* = 1; Russian & Bulgarian: *n* = 1; Ukrainian & Spanish; *n* = 1).

Thus, the final sample included 389 respondents (Austria: *n* = 100; Germany: *n* = 289). Table 2 shows the demographic statistics for the sample split by the host country and by status.

**Table 2:** Demographic information on the participants per country (Austria vs. Germany) per status (Migrants vs. Refugees).

		Austria (*n* = 100)		Germany (*n* = 289)	
		Migrants (*n* = 44)	Refugees (*n* = 56)	Migrants (*n* = 51)	Refugees (*n* = 238)
Gender	Female	31	53	44	205
	Male	13	3	6	32
	Wish not to disclose		0	1	1
Age	M (SD) MIN – MAX	37 (10) 21–55	42 (9) 19–62	36 (9) 21–60	40 (9) 18–71
Education	Middle school			0	4
	High school	4	2	2	10
	Vocational school	4	9	3	31
	BA	8	8	17	42
	MA/specialist Diploma	24	31	25	145
	PhD	4	9	3	6
Length of residency in the host country in months	M (SD) MIN – MAX	110 (83) 8–296	5 (2) 1–7	134 (115) 8–449	4 (2) 0–6
Language of the completed questionnaire	Russian	16	11	23	77
	Ukrainian	28	45	28	161

**Figure 1:**
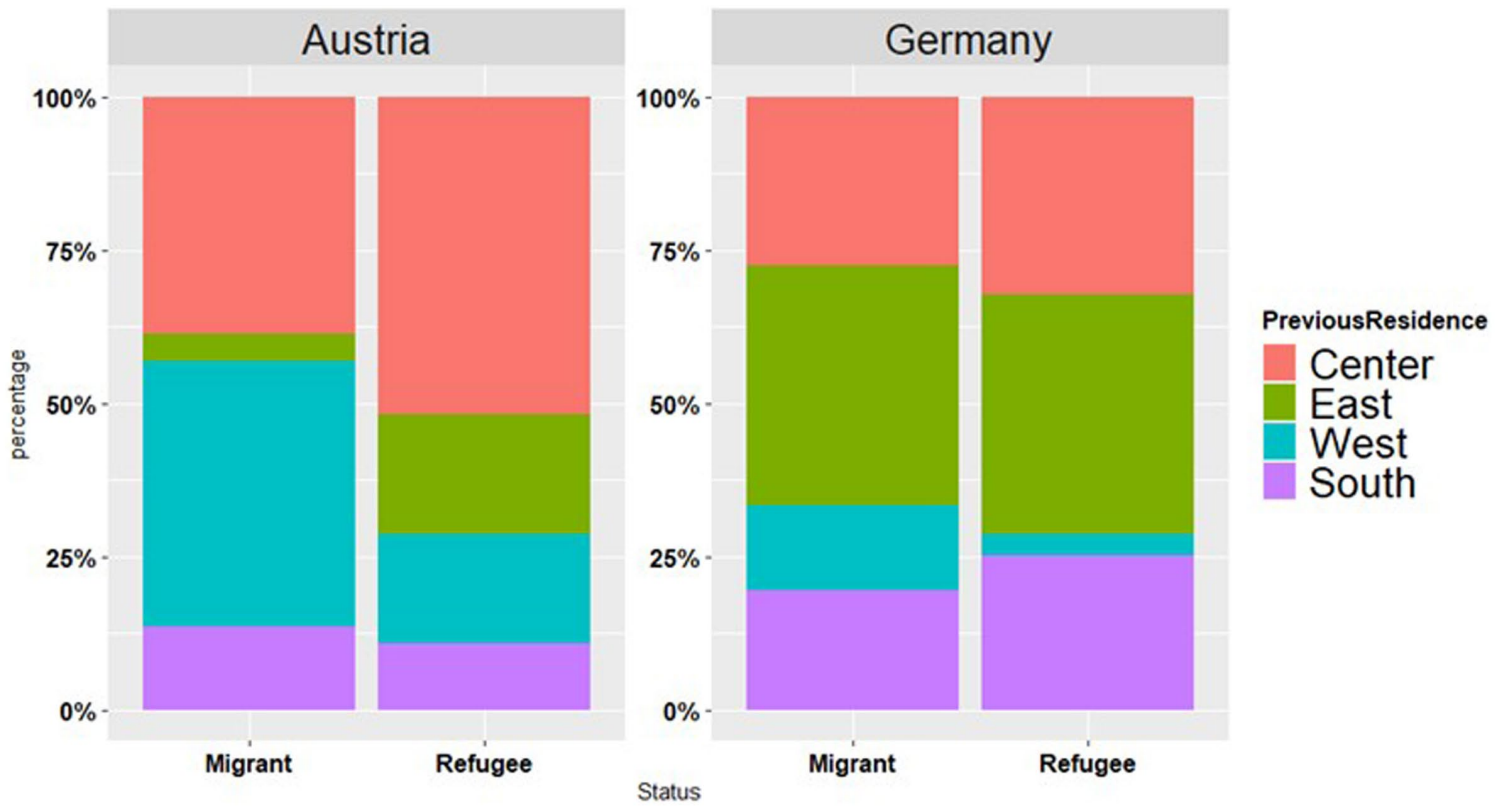
Previous residence in Ukraine reported by Austria and Germany-based participants.

**Figure 2:**
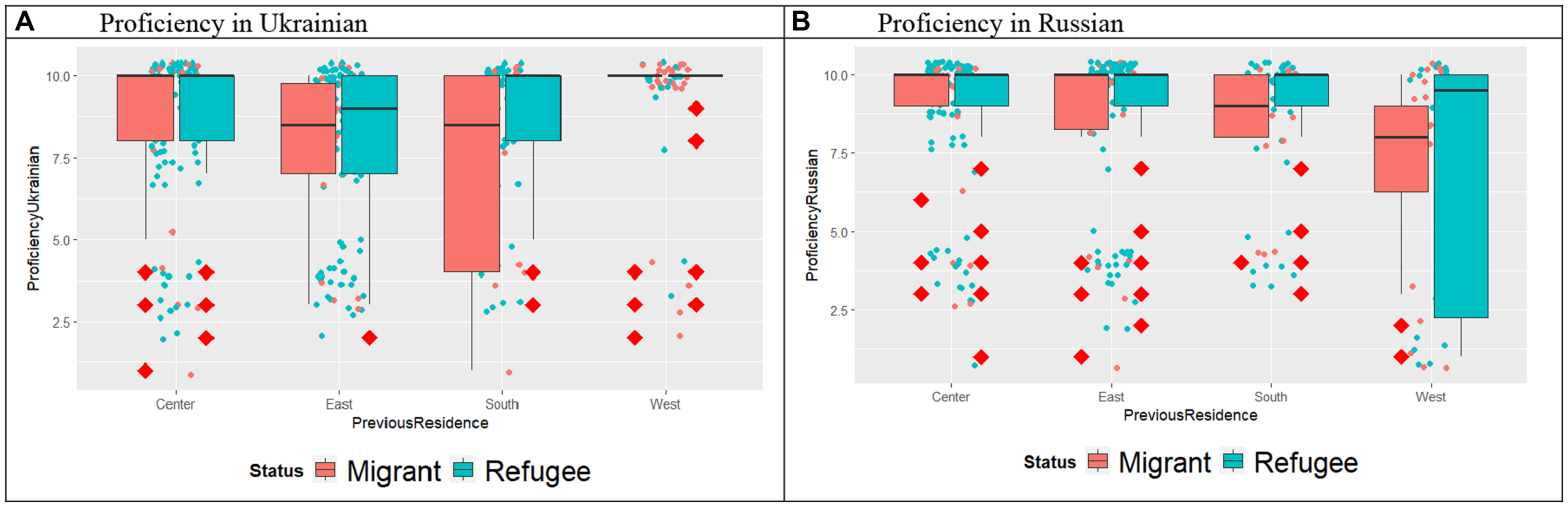
Self-reported language proficiency ratings in Ukrainian and in Russian of participants per Status per previous residence in Ukraine (Center, East, South, West).

Most of our Germany-based participants originated from the eastern parts of Ukraine (*n* = 113, 39%), the second largest group was from the central areas (*n* = 91, 31%), followed by the groups from the South (*n* = 70, 24%) and western parts (*n* = 15, 5%). By contrast, the “Eastern Ukrainian” group was one of the smallest one in the Austrian dataset (*n* = 13, 13%) and the South (*n* = 12, 12%) with most of the participants coming from central (*n* = 46, 46%) and western Ukraine (*n* = 29, 29%). The split per country per status is presented in Figure 1. The previous place of residence in Ukraine might be related to proficiency in the Russian and Ukrainian languages, as further visualized in Figures 2A,B.

**Figure 3:**
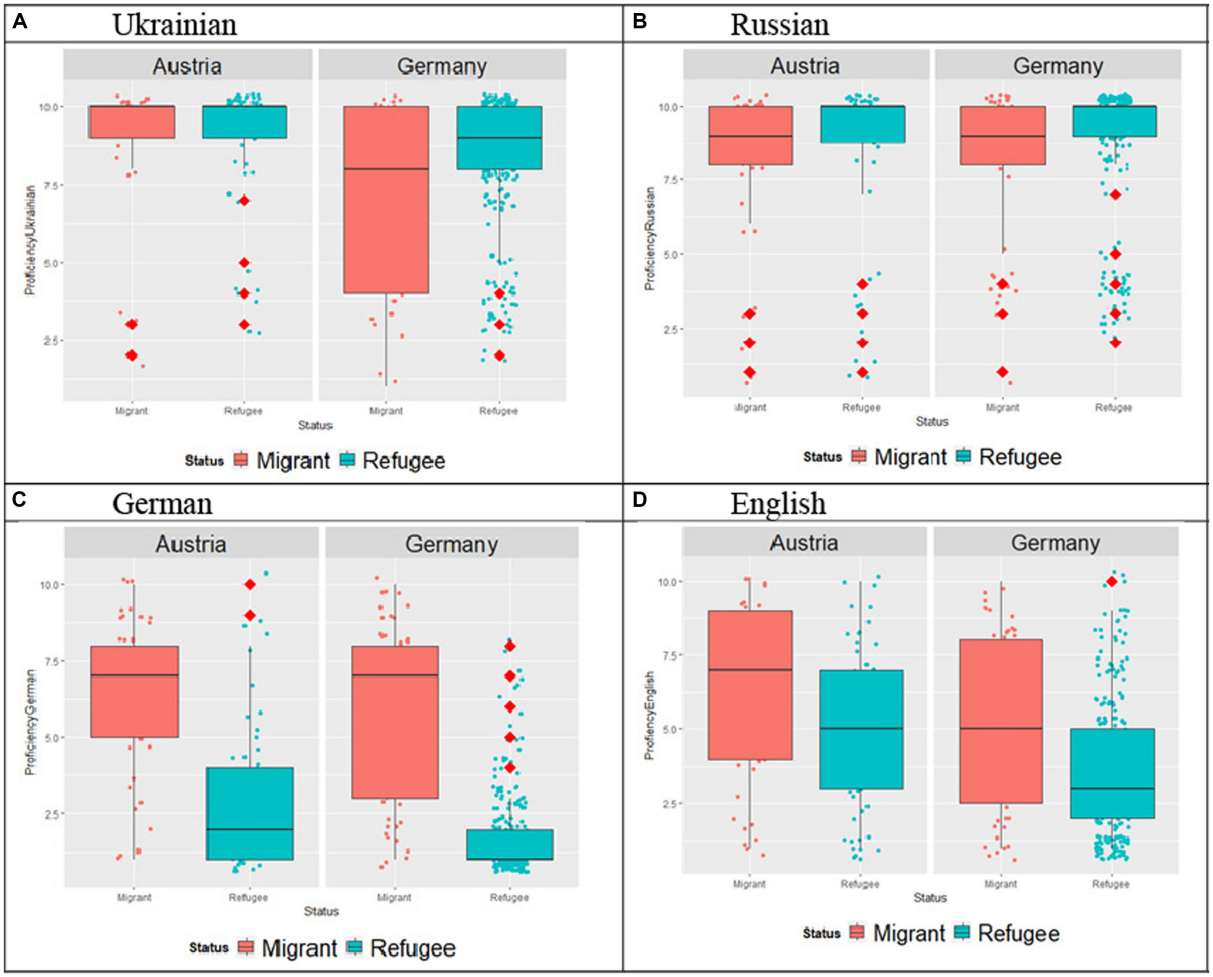
**(A–D)** Self-reported language proficiency ratings of participants per Language per Country per Status.

We asked people to provide self-reported language proficiency ratings in Ukrainian, Russian, German and English (see Figures 3A–D). This has been done to obtain a more holistic picture of self-reported language proficiency skills in multiple languages. These differences in the region of origin are also reflected in how participants from the two countries rated their language skills. We applied separate two-way ANOVAs with Country and Status as independent variables to analyze the data for each language. The results for the Ukrainian language showed an effect of Country: the participants in Austria reported its higher proficiency (*M* = 9.08, SE = 0.23) compared to the participants in Germany (*M* = 7.82, SE = 0.18), no effect of Status and no Country*Status interaction was detected. For Russian, all participants reported high ratings, there was no effect of Country, no effect of Status and no Status*Country interactions. Turning to the German self-rated proficiency, as expected there was an effect of Status, with Migrant participants reporting its proficiency (*M* = 6.14, SE = 0.21) than Refugees (*M* = 2.31, SE = 0.15), as well as an effect of Country, with participants in Austria reporting higher levels of proficiency (*M* = 4.67; SE = 0.20) than those in Germany (*M* = 3.79; SE = 0.15). Finally, similar to language proficiency in Germany, for proficiency in English there was an effect of status, with migrant participants reporting higher ratings (*M* = 5.65, SE = 0.27) than Refugees (*M* = 4.39, SE = 0.19), as well as an effect of Country, with participants in Austria reporting higher ratings (*M* = 5.65; SE = 0.20) than those in Germany (*M* = 4.39; SE = 0.20).

Furthermore, we evaluated whether the language proficiency of the participants varied in relation to the place of previous residence (see Figures 2A–D). We ran two-way ANOVAs with Previous Residence and Status as independent variables. As for the level of Ukrainian, the results indicated an effect of Previous Residence, no effect of Status, and no Status*Previous Residence interaction. Post-hoc comparisons revealed significant difference only between “East” (*M* = 7.85; SE = 0.27) and “West” (*M* = 9.04; SE = 0.36) subgroups (*p* = 0.0474), with the participants immigrating from “West” scoring higher than the participants from “East.” For Russian ratings, similarly to Ukrainian ratings there was an effect of Previous Residence, no effect of status and no Previous Residence*Status interaction. Post-hoc tests showed that the participants from “West” (*M* = 7.01; SE = 0.38) rated their proficiency in Russian significantly lower compared to the participants from other regions, the “East” (*M* = 8.39, SE = 0.29), “Center” (*M* = 8.75; SE = 0.26), and “South” (*M* = 8.69; SE = 0.35) (all comparisons at *p* < 0.05). No effect of previous residence was detected for the self-rated proficiency in German and in English.

**Figure 4:**
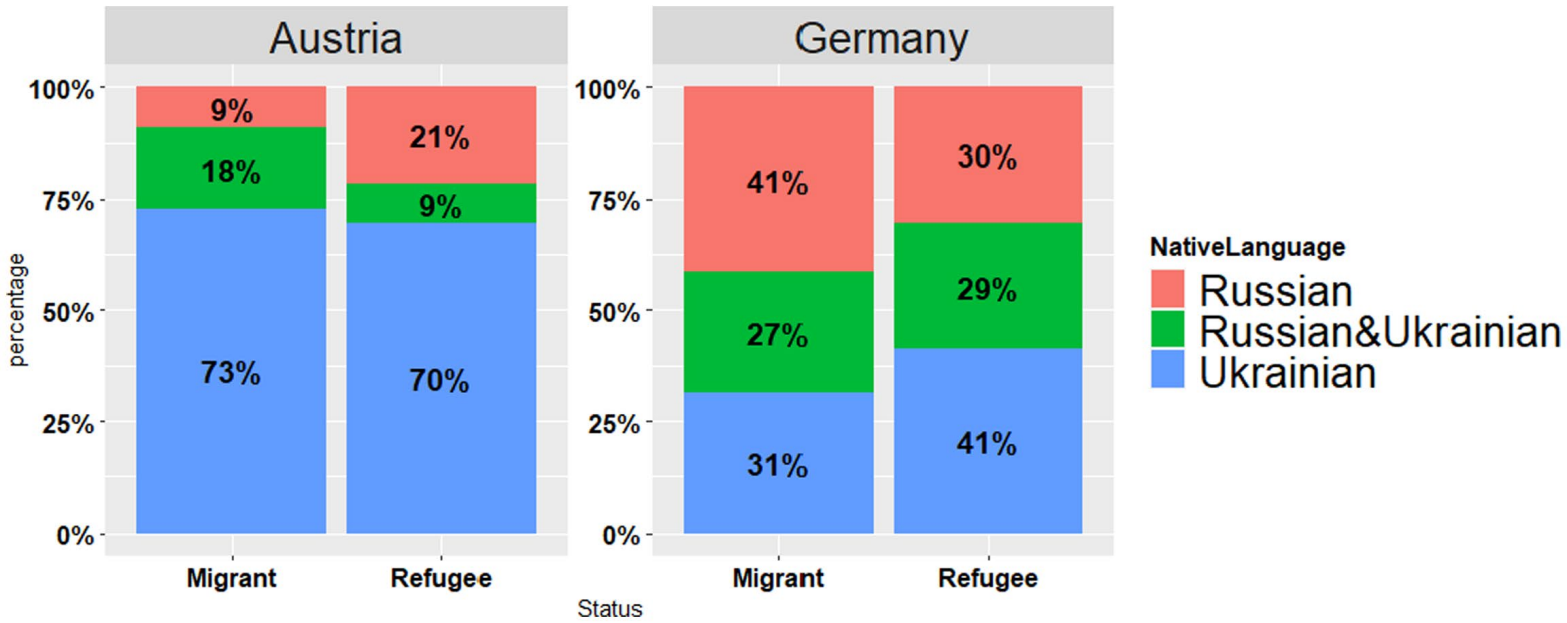
Reported native language of participants per country (Austria vs. Germany) per status (Migrant vs. Refugee).

Participants were asked to name their native language (see Figure 4). Most of the participants in the Austrian sample responded that either Ukrainian (Migrants: *n* = 32, 73%, Refugees: *n* = 39, 70%) or Ukrainian and Russian were their native languages (Migrants: *n* = 8, 18%, Refugees: *n* = 5, 9%), while a small fraction considered Russian to be their native language (Migrants: *n* = 4, 9%, Refugees: *n* = 12, 21%). The picture was slightly different for the German sample, either Ukrainian (Migrants: *n* = 16, 31%, Refugees: *n* = 98, 41%) or Ukrainian and Russian (Migrants: *n* = 14, 27%, Refugees: *n* = 68, 29%) were noted as native languages, whereas a sizable portion responded that Russian was their native language (Migrants: *n* = 21, 41%, Refugees: *n* = 72, 30%).

**Figure 5:**
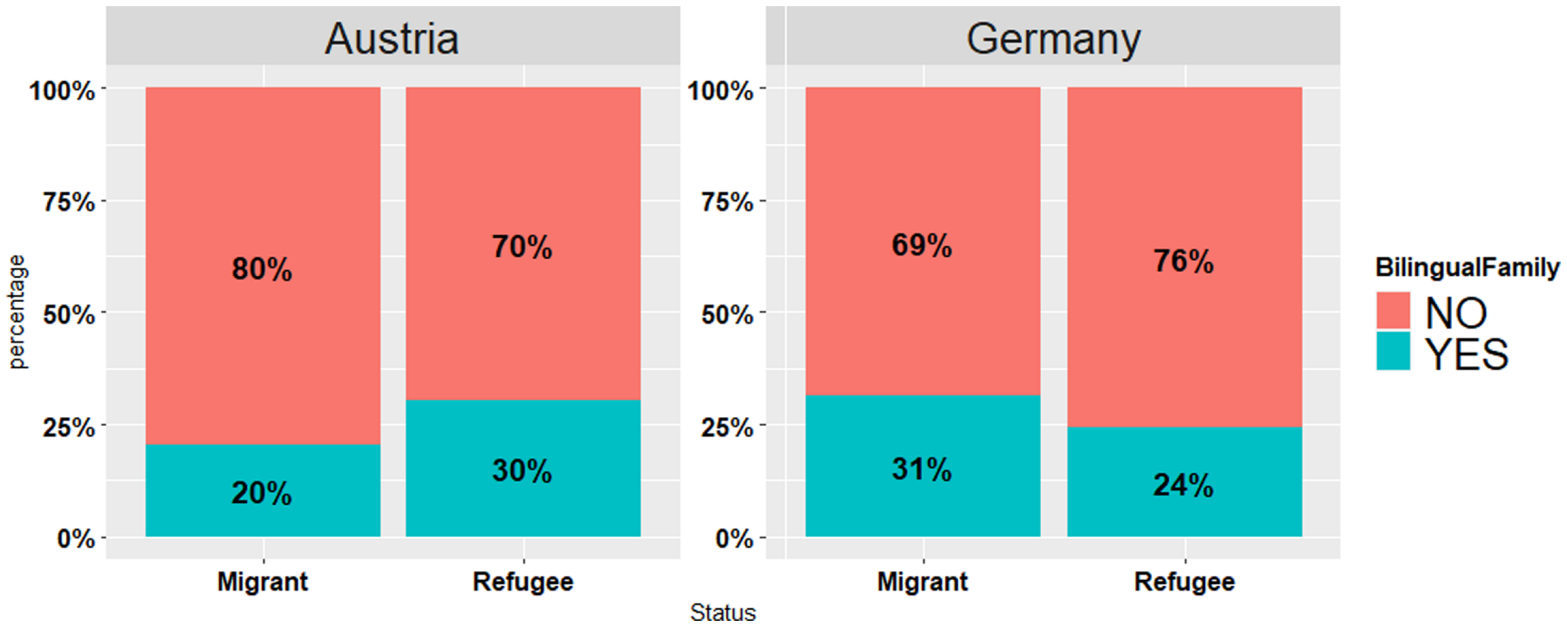
Responses to the question “Do you come from a bilingual family?” per country (Austria vs. Germany) per status (Migrant vs. Refugee).

Furthermore, participants were asked to provide information on the linguistic status of their families (see Figure 5). In both samples, a minority of individuals indicated they hailed from bilingual families: Austria (Migrants: *n* = 9, 20%, Refugees: *n* = 17, 30%) and Germany (Migrants: *n* = 16, 31%, Refugees: *n* = 58, 24%).

### 4.3 Language use and identity

#### 4.3.1 Language use in Austria and Germany

**Figure 6:**
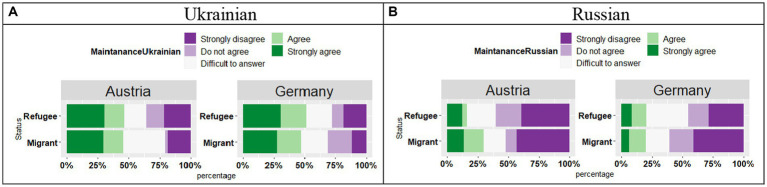
**(A,B)** Responses for “There are few opportunities to support this language in the host country” by Country and by Status.

The participants in the Migrant and Refugee subgroups in Austria and Germany responded similarly to the question about the opportunities to support language knowledge: more participants agree that the knowledge of Ukrainian is harder to support than Russian (see Figures 6A,B). For the maintenance of Ukrainian: the ordinal logistic regression showed no effect of Country (*t* = −0.17, *p* = 0.43), no effect of Status (*t* = 0.46, *p* = 0.32); and no interaction (*t* = 0.55, *p* = 0.29). For the maintenance of Russian, similarly no effect of Country (*t* = 0.71, *p* = 0.48), no effect of Status (*t* = 0.64, *p* = 0.52); and no interaction (*t* = 1.49, *p* = 0.14).

**Figure 7:**
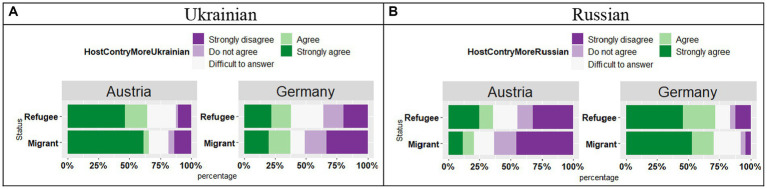
**(A,B)** Responses for “I use more of this language in the host country” by Country and by Status.

Overall, the participants answered that there were and are few possibilities to use Ukrainian (Figures 7A,B), yet there were differences in responses provided by the participants in Austria and Germany with respect to the use of Ukrainian, as shown by the effect of Country (*t* = 4.41, *p* < 0.001) in the absence of the Status effect (*t* = 0.84, *p* = 0.40) and Country*Status interaction (*t* = 1.52, *p* = 0.13). In Austria, the respondents were more likely to strongly disagree with this statement regarding the opportunities to use Ukrainian compared to the participants in Germany. Turning to the use of Russian, differences in the responses across the two countries were observed (*t* = 5.88, *p* < 0.001), with a marginal effect of Status (*t* = 2.13, *p* = 0.05) and significant Country*Status interaction (*t* = 2.13, *p* = 0.03). Follow-up analyses on the significant Groups*Status interactions indicated differences in the responses between Migrant and Refugee groups in Austria (*p* = 0.048), yet no differences between these two subgroups in Germany (*p* = 0.35): Migrant participants in Austria were more likely to respond that it is not the case that they are more opportunities to use Russian in Austria.

#### 4.3.2 Language knowledge

**Figure 8:**
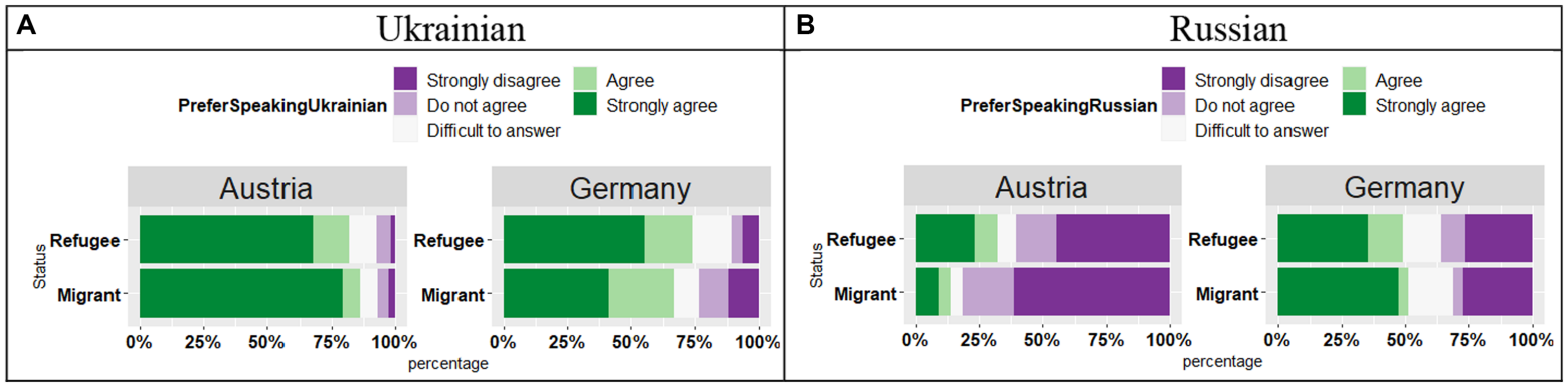
**(A,B)** Responses for “I prefer using this language” by Country and by Status.

The responses from respondents in Austria and Germany differed regarding their preferences for using Ukrainian (see Figure 8A), as shown by the effect of Country (*t* = 3.63, *p* < 0.001) and Country*Status interaction (*t* = 2.04, *p* = 0.04), in the absence of the Status effect (*t* = 1.21, *p* = 0.23). In the German sample, Refugees were more likely to respond that they prefer speaking Ukrainian, while in the Austrian sample more Migrant participants tended to answer that they preferred speaking Ukrainian.

With respect to their preferences for using Russian (see Figure 8B), there was an effect of Country (*t* = 4.58, *p* < 0.001), an effect of Status (*t* = 2.09, *p* = 0.04) and a significant Country*Status interaction (*t* = 2.19, *p* < 0.03). In Germany, both Refugee and Migrant groups were more likely to provide “Agree” and “Strongly agree” responses, while in Austria both groups were more likely to provide “Disagree” and “Strongly disagree” responses. Furthermore, these trends were stronger in the migrant groups.

**Figure 9:**
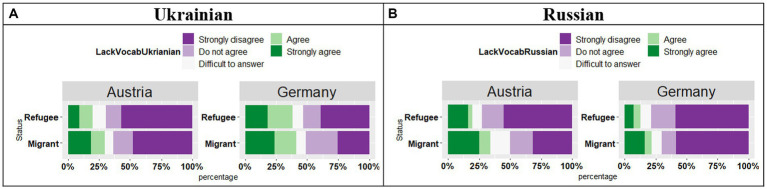
**(A,B)** Responses for “Sometimes I feel that I lack vocabulary when I speak in this language.”

The participants were asked about the lacking vocabulary in Ukrainian and Russian (see Figures 9A,B). Both groups in both countries provided similar responses regarding Ukrainian: the participants generally disagreed with the statement. With respect to the preference for Russian, there was an effect of Country (*t* = 4.58, *p* < 0.001), an effect of Status (*t* = 2.09, *p* = 0.04) and a significant Country*Status interaction (*t* = 2.19, *p* = 0.03). In Austria and Germany, both Refugee and Migrant groups were more likely to provide “Strongly disagree” responses, yet in Austria the trend was somewhat less strong in the Migrant group.

#### 4.3.3 Emotional connection

**Figure 10:**
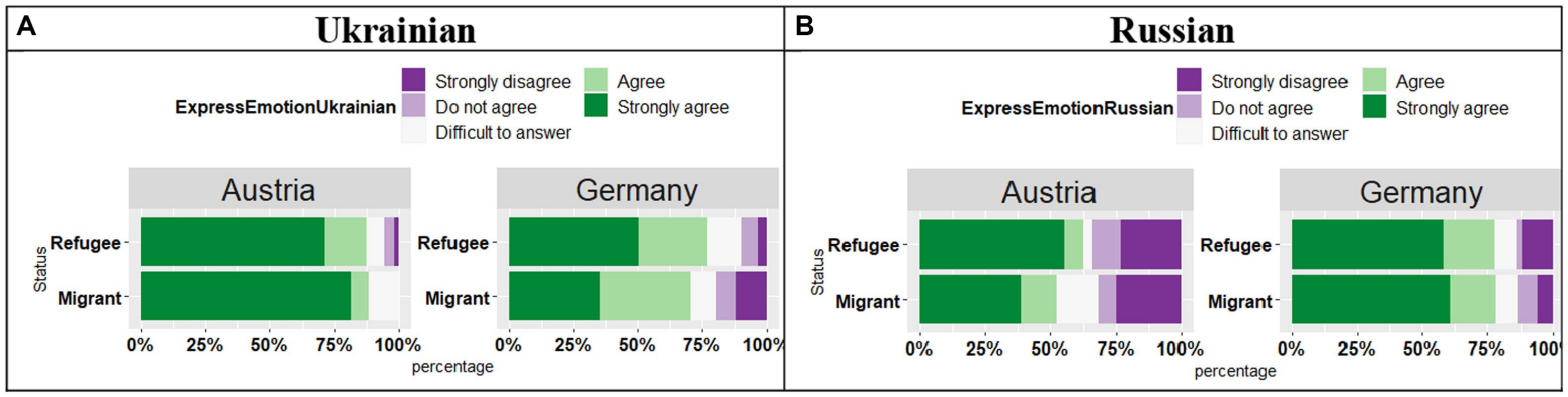
**(A,B)** Responses for “I feel I can express my emotions in this language.”

The participants were asked about their emotional connection to the two languages (Figures 10A,B). The analysis of the responses “I feel I can express my emotions in this language.” in Ukrainian revealed an effect of Country (*t* = 4.20, *p* < 0.001), no effect of Status (*t* = 1.16, *p* = 0.24), yet a significant Country by Status interaction (*t* = 2.02, *p* = 0.04), which means that in Austria the participants were more likely to “Strongly agree” with the statement, whereas in Germany they were more likely to provide the agree answer “Agree.” And these responses were reversed in the Refuge and Migrant groups. As for expressing emotions in Russian, the analysis revealed an effect of Country (*t* = 2.63, *p* = 0.01), no effect of Status (*t* = 1.27, *p* = 0.21), and no significant Country by Status interaction (*t* = 1.24, *p* = 0.21), meaning that in Germany the respondents were more likely to select “Strongly agree” and “Agree” answers compared to respondents in Austria.

**Figure 11:**
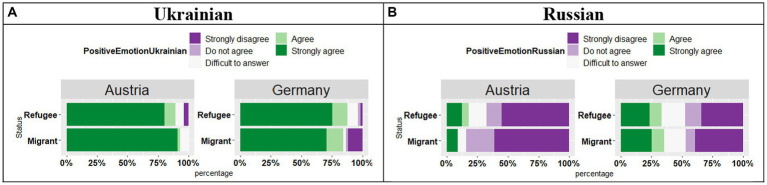
**(A,B)** Responses for “This language evokes positive emotions in me.”

The analysis of the responses *“This language evokes positive emotions in me”* in Ukrainian (see Figure 11A) revealed an effect of Country (*t* = 2.43, *p* = 0.01), no effect of Status (*t* = 1.43, *p* = 0.15), and no significant Country by Status interaction (*t* = 1.70, *p* = 0.09), signifying that in Austria the participants were more likely to “Strongly agree” with the statement, whereas in Germany they were more likely to select the answer “Agree.”

As for the positive emotions in Russian (see Figure 11B), in contrast to Ukrainian, in both countries the participants provided mainly “Strongly disagree” and “Disagree” responses regardless of the participants` Status. Our analysis showed an effect of Country (*t* = 3.07, *p* < 0.01), no effect of Status (*t* = 1.00, *p* = 0.31), and no significant Country by Status interaction (*t* = 0.73, *p* = 0.46), meaning that negative responses were stronger in Austria than in Germany.

**Figure 12:**
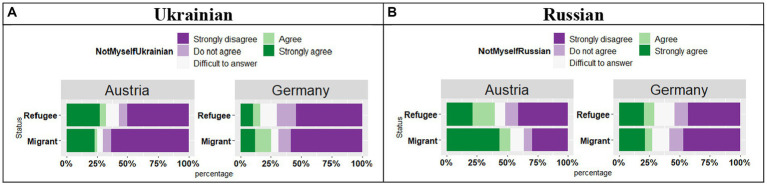
**(A,B)** Responses for “I do not feel like myself when I speak this language.”

The analysis of the responses “*I do not feel like myself when I speak this language*” in Ukrainian (see Figure 12A) revealed no effect of Country (*t* = 0.17, *p* = 0.86), no effect of Status (*t* = 1.42, *p* = 0.16), and no significant Country by Status interaction (*t* = 1.09, *p* = 0.27), thus regardless of the country and regardless of the Status, the participants responses were similar: they strongly disagreed with the statement.

Turning to the parallel statement regarding Russian (see Figure 12B), the analysis showed a significant effect of Country (*t* = 2.55, *p* = 0.01), no effect of Status (*t* = 1.94, *p* = 0.05), and no significant Country by Status interaction (*t* = 1.77, *p* = 0.08). The participants in Austria were more likely to agree with the statement as compared to the ones in Germany.

#### 4.3.4 Identity

**Figure 13:**
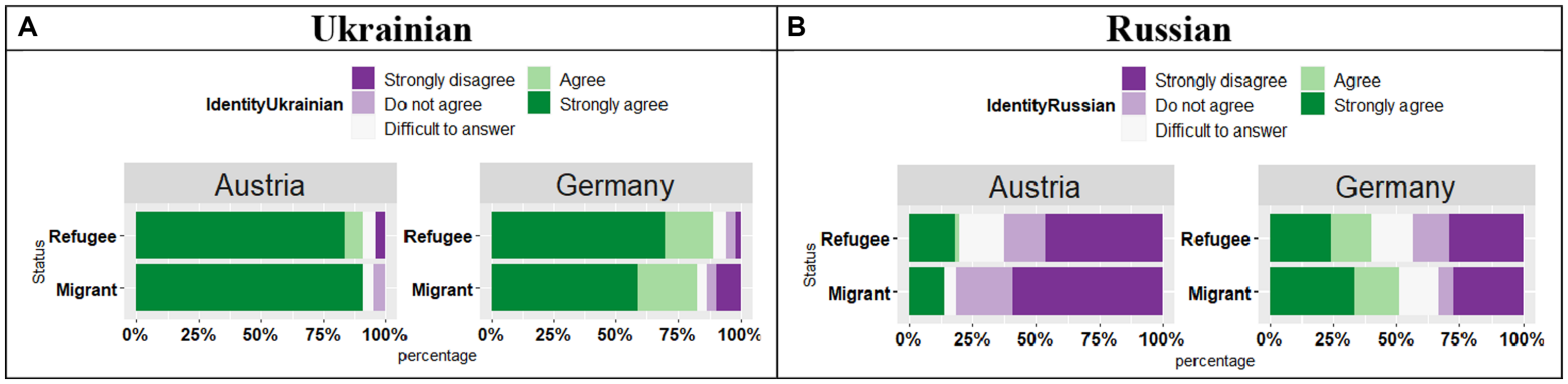
**(A,B)** Responses for “Knowing this language is an important part of my identity.”

The analysis of the responses “*Knowing this language is an important part of my identity*” in Ukrainian (see Figure 13A) revealed a significant effect of Country (*t* = 4.30, *p* < 0.01), no effect of Status (*t* = 1.16, *p* = 0.24), yet a significant Country by Status interaction (*t* = 2.01, *p* = 0.04). Overall, the participants in both countries provided a “Strongly Agree” response, however, this trend again was stronger in Austria compared to Germany. There were differences between the responses of Migrant and Refugee participants in Austria and Germany, with Refugee patricians in Germany providing “Strongly agree” responses more frequently than Migrant participants in Germany.

With regard to the Russian language as an important part of the participants’ identity (see Figure 13B), the results showed a significant effect of Country (*t* = 4.24, *p* < 0.01), no effect of Status (*t* = 1.49, *p* = 0.24), yet a significant Country by Status interaction (*t* = 1.96, *p* = 0.04). The participants in Austria were more likely to strongly disagree with the statement compared to the participants in Germany. In Germany the Migrant group was more likely to select “Strongly agree” and “Agree” statements compared with the respondents in Austria.

**Figure 14:**
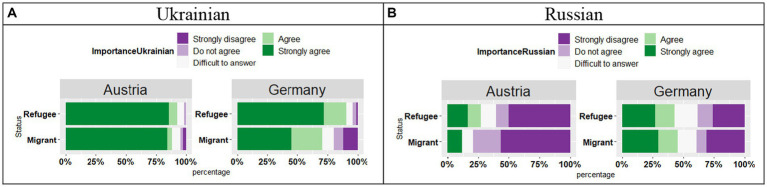
**(A,B)** Responses for “It is important for me that my children know this language.”

The analysis of the responses “*It is important for me that my children know this language*” for Ukrainian (see Figure 14A) revealed a significant effect of Country (*t* = 3.82, *p* < 0.01), no effect of Status (*t* = 0.31, *p* = 0.75), and no significant Country by Status interaction (*t* = 1.67, *p* = 0.09). Overall, the participants in both countries selected a “Strongly Agree” response, however, this trend again was stronger in Austria as compared to Germany. Furthermore, while in Austria, no difference was observed between Migrant and Refugee participants, in Germany the Migrant participants slightly differed from the Refugee participants, as they were less likely to select a “Strongly agree” response.

The analysis of the responses “*It is important for me that my children know this language*” for Russian (see Figure 14B) revealed a significant effect of Country (*t* = 3.57, *p* < 0.01), no effect of Status (*t* = 1.30, *p* = 0.19), and no significant Country by Status interaction (*t* = 1.03, *p* = 0.30). While in Austria both groups strongly disagreed with the statement for Russian, in Germany, the responses were split between “Strongly disagree/Disagree” and “Strongly Agree/Agree.”

#### 4.3.5 Language attitude change

**Figure 15:**
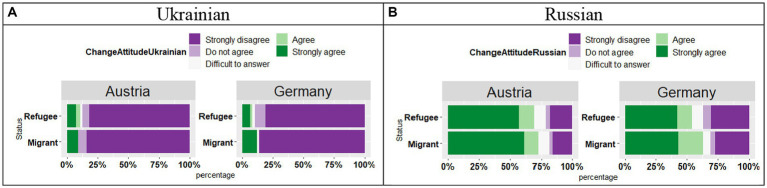
**(A,B)** Responses for “The war and the political events of recent years have changed my attitude towards the language for the worse.”

The analysis of the responses “*The war and the political events of recent years have changed my attitude towards the language for the worse.*” for Ukrainian (see Figure 15A) showed no significant effect of Country (*t* = 0.18, *p* = 0.85), no effect of Status (*t* = 0.25, *p* = 0.80), and no significant Country by Status interaction (*t* = 0.20, *p* = 0.84). The participants in both countries, irrespective of their Status selected a “Strongly disagree” response.

As for Russian (see Figure 15B), the picture was different: the most common responses were “Strongly agree” and “Agree.” The analysis showed that these responses did not vary per Country and per Status, as there was no significant effect of Country (*t* = 1.70, *p* = 0.09), no effect of Status (*t* = 0.42, *p* = 0.67), and no significant Country by Status interaction (*t* = 0.03, *p* = 0.97).

**Figure 16:**
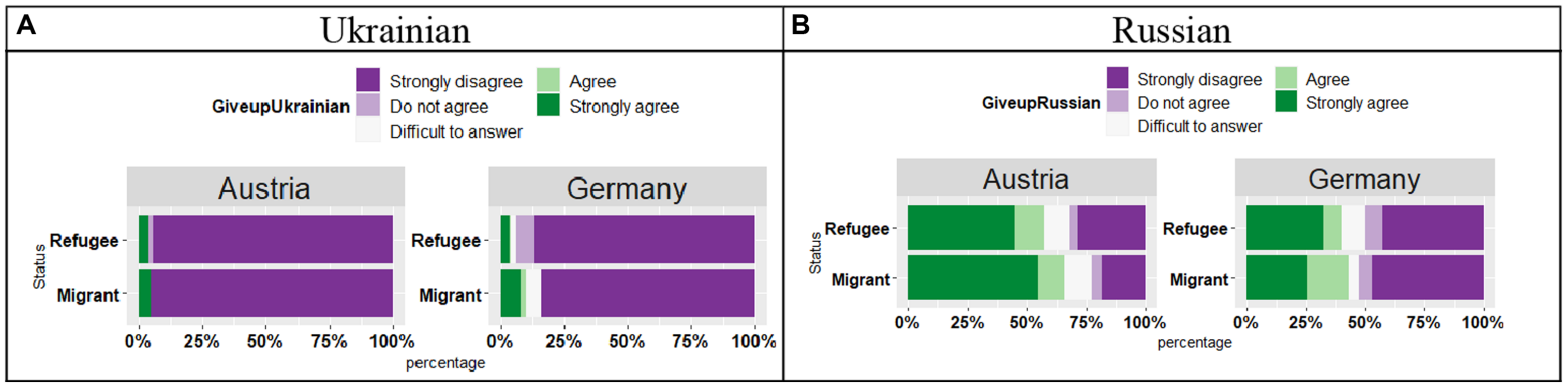
**(A,B)** Responses for “I would like to stop using this language altogether.”

As for Russian (see Figure 16B), the analysis showed a significant effect of Country (*t* = 3.10, *p* < 0.01), no effect of Status (*t* = 1.12, *p* = 0.26), and no significant Country by Status interaction (*t* = 1.23, *p* = 0.22). the differences were visible for the “Strongly disagree” pattern of responses, while in German this response made up around 50% of responses; in Austria this pattern was observed in only approximately 25% of responses.

In conclusion, the results indicated that the effect of Status (Migrant, Refugee) was not significant for most of the statements examined in the survey. The effect of Country (Austria, Germany) was significant for most of the statements: the respondents in Germany were less categorical against the Russian language as compared to the respondents in Austria.

### 4.4 Language attitudes and (socio)-linguistic background factors: a network analysis

**Figure 17:**
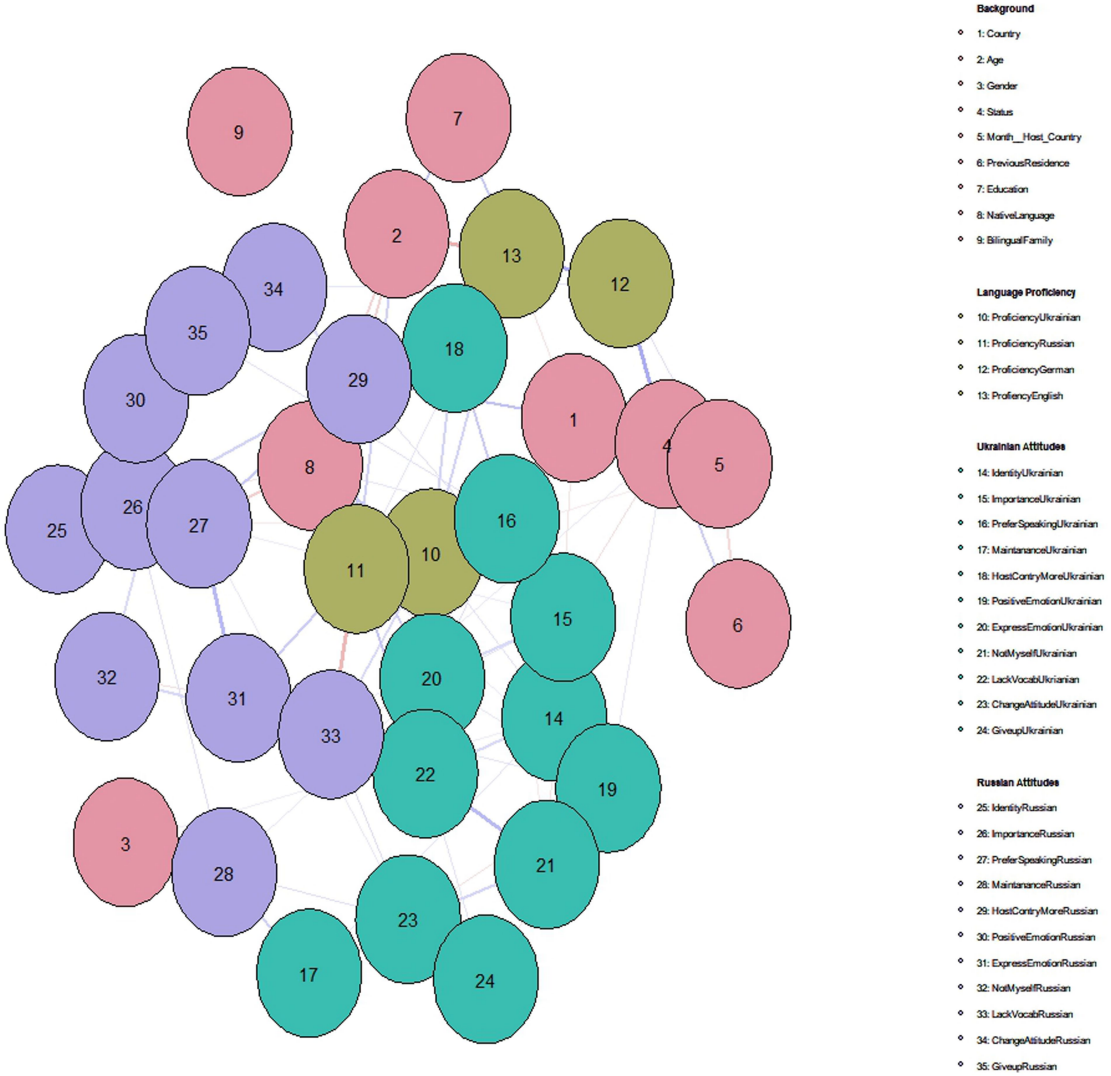
A network model of the attitudes towards Ukrainian and Russian, individual background factors and language proficiency levels of the participants. The strength of the relationship between the nodes is indicated by line thickness and color density: the thicker the line, the stronger the relationship. Positive relationships are purple, negative relationships are red.

In our subsequent analysis, we evaluated the links between the participants’ background information, his/her language proficiency and his/her attitudes towards Ukrainian and Russian. For these purposes, we conducted a network analysis (see Figure 17). Network modeling proves invaluable in evaluating intricate, dynamic, and multivariate systems that may not be adequately elucidated using one-way statistical approaches (Zalbidea et al., 2023). Moreover, this approach serves as an effective tool for exploratory analysis, facilitating the generation of hypotheses through estimated relationships and interdependencies (Epskamp et al., 2018). Comparatively, it is argued to be better suited for exploration than methodologies like structural equation modeling, as discussed by Abacioglu et al. (2019). Network models comprise nodes, which depict the variables included in the model, and edges that link these nodes, representing partial correlation coefficients among the variables (Bringmann et al., 2019). The density and color of edges can vary, indicating the strength and direction (positive or negative) of relationships, respectively. It is essential to recognize that although network models offer insights into partial correlations among variables, they do not imply causality. We employed the Gaussian Graphical Model (GGM) alongside Spearman partial correlations, enabling us to accommodate a combination of categorical and continuous variables within our analysis (see Epskamp and Isvoranu, 2022).

Two background factors were found not to be related to language use and language attitudes: gender and bilingual family status. The results demonstrate a positive interconnection among Ukrainian attitudes (green nodes) and similarly among Russian attitudes (purple nodes). Additionally, proficiency in the Ukrainian language is directly associated with positive attitudes toward Ukrainian. Furthermore, native language (code as: Ukrainian = 3, Ukrainian & Russian = 2, Russian = 1) is negatively related to attitudes to Russian, yet positively related to Ukrainian proficiency and attitudes to Ukrainian. Interestingly, the proficiency levels in German and English are not directly correlated with attitudes toward Ukrainian and Russian. However, it is not surprising that proficiency in these languages correlates with the participants’ status (Refugee or Migrant) and their length of residency in the host country. Migrant participants reported higher proficiency levels in German and English compared to refugees.

**Figure 18:**
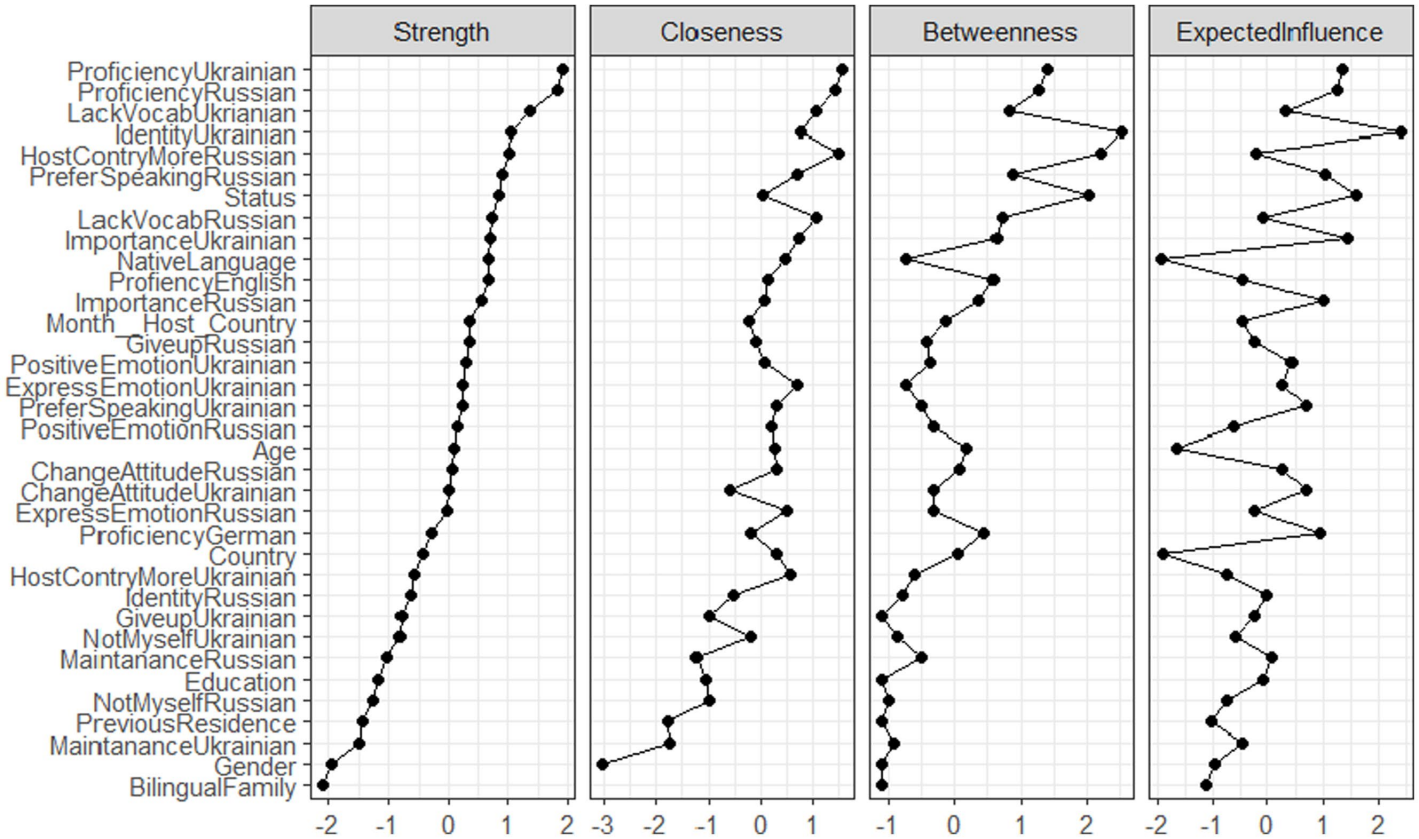
Centrality plot (strength, closeness, betweenness and expected influence) for the network of the attitudes towards Ukrainian and Russian, individual background factors and language proficiency levels of participants.

Furthermore, we also estimated the stability of centrality measures of our network (see Figure 18). The commonly used centrality metrics encompass node strength, which signifies the total number of connections a node possesses along with their robustness; betweenness, indicating how frequently a node lies on the shortest path between two other nodes; and closeness, signifying the proximity of the node to others, along with the anticipated impact each node carries within the network (Zalbidea et al., 2023). In terms of strength, proficiency in Ukrainian and in Russian exhibited the highest strength, whereas the bilingual status of the family in which the participant was born had the lowest strength in the model. Based on the index of Expected Influence, “Knowing Ukrainian is an important part of my identity” demonstrated the highest influence on the nodes of the network.

## 5 Discussion

The paper presented the current attitudes of diasporic Ukrainian communities (migrants vs. refugees) in Austria and Germany towards the Ukrainian and the Russian languages. By looking into differences and similarities in the language use and attitudes, the study aimed to investigate to what extent the granted symbolic and pragmatic status of Ukrainian vs. Russian is associated with the (socio) linguistic background of the respondents.

Based on previous research into the attitudes of Ukrainians towards the Ukrainian and Russian languages in Ukraine (cf. Hentschel and Zeller, 2016; Kulyk, 2023), we assumed that there would be an increase of the symbolic status of Ukrainian in the diasporic communities and, as a consequence, a (planned or aspired) increase of the use of Ukrainian in migration-affected multilingual settings. With regard to the sustainability of Ukrainian–Russian bilingualism, we also expected that despite a higher pragmatic value of Russian, which functions as a *lingua franca* in post-Soviet (diasporic) communities, Ukrainian will be not replaced by Russian in everyday use due to its increasing symbolic value. Our study confirmed these hypotheses.

Starting with the socio-linguistic background data of the respondents, there were differences in the levels of (self-evaluated) language proficiency in Ukrainian (see Figures 3A), with Austrian respondents reporting higher proficiency in Ukrainian than German ones; yet no differences were found between the groups with regard to proficiency in Russian. It should be noted that most of our Germany-based participants were from the regions with Russian-dominant bilingualism of Ukraine (see Figure 1). By contrast, in the Austrian dataset the eastern Ukrainian group was one of the smallest ones, with the most representative group coming from the regions with predominantly Ukrainian-dominant bilingualism (see Figures 1, 4). The differences in the proficiency in Ukrainian were further reflected in the place of previous residence (see Figure 2A): the participants coming from “West” reported higher proficiency in Ukrainian than the participants from “East,” whereas the same respondents from the “West” rated their proficiency in Russian significantly lower as compared to the participants from other regions. Thus, self-reported levels of Ukrainian and Russian language proficiency varied by the place of origin (“West” vs. “East”) and their subsequent place of residence, i.e., Austria vs. Germany. The level of language proficiency was also reflected in the respondents’ answers to the question which language they considered to be their native language. In Austria, the majority of respondents said that Ukrainian was their native language, whereas in the German sample, the responses were roughly equally split between the three options (i.e., Ukrainian, Ukrainian and Russian, Russian) (see Figure 4). Despite high levels of reported language proficiency in both languages, only a small percentage of the respondents said that they were raised in bilingual families (see Figure 5). Accordingly, the differences between the (socio) linguistic characteristics of the German vs. Austrian diasporic communities corelate with the reported language proficiency and, correspondingly, with the reported role of language proficiency in the speaker’s identity.

Thus, we identified the key role of subjective language proficiency in the reported attitudes towards the respondents’ language identity; this language proficiency, in turn, is linked to their region of origin in the homeland.

Concerning the transfer of post-colonial Ukrainian–Russian bilingualism from the homeland to the host country, our study shows the following picture: most participants in the Austrian sample identified Ukrainian as their native language while a substantial group in the German sample identified Russian as their native tongue. Accordingly, the participants in Austria reported higher proficiency in Ukrainian as compared to the participants in Germany. However, for Russian, all the participants in both countries reported high ratings. Thus, we have verified that even though only a small part of our respondents reported being from a Ukrainian–Russian bilingual family, they have access to and use both languages to varying degrees. From the responses, a well-established societal (not familial) Ukrainian–Russian bilingualism in the homeland can be assumed, at least in the first generation of migrants and refugees. Whether and to what extent Ukrainian–Russian bilingualism can and will be maintained in the diasporic communities, especially in the subsequent generations, remains a topic of future investigations.

The study aimed to evaluate the granted symbolic and pragmatic status of Ukrainian and Russian in the Ukrainian migrant and refugee communities in both countries. Starting with the symbolic value of the two languages, their status has changed in the context of war, (cf. Kulyk, 2023; Racek et al., 2024). Even the choice of the language (Ukrainian vs. Russian) in which the participants filled out the questionnaires was symptomatic (see Table 2). The majority of the respondents chose to reply in the Ukrainian language, including refugees of eastern Ukrainian origin in Germany, i.e., from the regions with Russian-dominant bilingualism. Our randomly generated groups of respondents, i.e., people who were spontaneously willing to fill out our anonymous survey correlate with the general regional characteristics of migrants and refugees and their present distribution between both host countries (the majority of them came from eastern Ukraine, and predominantly settled in Germany). Interestingly, there was no effect of migrant status (Migrant vs. Refugee) for any of the questions directly or indirectly tapping into the symbolic value of the two languages.

In terms of the granted symbolic and pragmatic value of both languages, we identified the following trends linked to the reported language attitudes and the (socio) linguistic background of respondents.

The main point of the discussion is whether and if so to what extent the reported symbolic value of languages (Russian and Ukrainian) is also representative in the context of migration. A positive symbolic value of Ukrainian and, respectively, a negative symbolic value of Russian come to the fore in the questions asking about emotional reactions evoked by both languages (see Figures 10A,B–12A,B), about individual and collective identity (see Figures 13A,B), and about an aspired transmission of the language to the next generation (Figures 14A,B). Thus, according to our findings, attitudes have changed or intensified with the onset of the military invasion, indicating a very strong and positive correlation. It should also be emphasized here, that our questions correspond with the main factors of language vitality. However, there is not necessarily a connection between language use and its (current) symbolic value.

With regard to the pragmatic value of Ukrainian and Russian, there seems to be a consensus on a higher pragmatic value of Russian in the diasporic communities with a Ukrainian background, especially in Germany, despite the stigmatized status of Russian and negative emotions that it may evoke. For example, the participants agreed that there are more opportunities to use Russian, and this is more evident in the German sample (see Figures 5A,B). With respect to language use, respondents in Germany seem to agree that they use even more Russian, whereas the respondents in Austria seem to use more Ukrainian (see Figures 6A,B). When looking at the preference (s) in the language use (see Figure 7A), again differences emerged between Austria and Germany, with a preference for Russian in the German sample. Thus, Germany-based migrants and refugees of eastern Ukrainian origin reported their targeted maintenance of Russian, also in subsequent generations. Unlike the symbolic value of the two languages which was not affected by the migrant status (Migrant vs. Refugee), the pragmatic value was related to the migration status. In Germany, both Refugee and Migrant groups were more likely to provide “Agree” and “Strongly agree” responses for the question regarding the use of Russian, while in Austria both groups were more likely to provide “Disagree” and “Strongly disagree” responses. Furthermore, these trends were stronger in the migrant groups. Also, with respect to lacking vocabulary in Russian, both Refugee and Migrant groups in Austria and in Germany were more likely to provide “Disagree” responses, meaning that their Russian proficiency does not show any signs of attrition. This trend was slightly different in the Migrant group in Austria, who seem to start showing the first signs of language attrition, which was reflected in less determined proficiency levels in Russian.

Thus, the correlation of the symbolic and pragmatic values of Ukrainian and Russian in the surveyed groups is imbalanced: Ukrainian has gained and is gaining a higher symbolic status, whereas Russian still possesses a higher pragmatic status despite its (nearly unanimously reported) negative symbolic status. In light of the imbalance between symbolic and pragmatic values of both languages, it is complicated to make well-founded predictions about the vitality of Ukrainian vs. Russian in the Ukrainian diasporic communities in Austria and Germany and the evolution of Ukrainian–Russian bilingualism. Based on our dataset, we expect that the documented high proficiency in Ukrainian and in Russian in the Ukrainian-dominant vs. Russian-dominant bilingual groups of the diasporic communities will further work as a key factor in language maintenance and vitality. At the same time, however, we expect that the increasing symbolic value of Ukrainian and the diminishing value of Russian will lead to an increase in the use of Ukrainian also in Russian-dominant speakers of Ukrainian migrants and refugees, even as an insider-code in hermetic minority groups.

Our network analysis showed that proficiency in Ukrainian and in Russian had the highest strength in the network, whereas the bilingual status of the family in which the participant was born had the lowest strength in the model. For example, “Knowing Ukrainian is an important part of my identity” is shown to have the highest influence on the nodes of the investigated network looking into language use and language attitudes. In the current study, the level of proficiency in Ukrainian seems to affect the language use, the language attitudes, and the identity of a person: the higher the level of self-rated proficiency in Ukrainian, the stronger the reported Ukrainian identity. Therefore, in this language dyad, identity and language proficiency seem to go hand in hand. However, in other communities, this is not necessarily the case. For example, the level of objective language proficiency in Hebrew (as measure by a naming task) did not correlate with identity indices for the Jewish English–Hebrew speaking community in the USA as evaluated through a network analysis (e.g., Fridman et al., 2024, submitted).

As a first exploration of Ukrainian–Russian bilingualism in the war-affected migrant communities, our study is not without limitations. Firstly, there is a need for verification of the reported self-rated language proficiency. Future studies on Ukrainian–Russian bilingualism in the diasporic context should include psycholinguistic tasks such as naming and/or narrative elicitation tasks in order to obtain reliable objective measures of language proficiency. In the framework of our study, we can (partly) verify the targeted and the real use of languages in our respondents. However, the actual implications of the reported targeted language use and an examination of the possible discrepancies between the speakers’ intentions and reality are to be investigated in a subsequent language data-based study. Secondly, for a more compelling investigation, larger-scale quantitative studies are needed. However, after the provided examination of the collected data, we believe that our random sample is quite representative within the general context of the war-affected diasporic communities. Because we are primarily interested in such linguistic issues as language variation in multilingual settings, we planned our study as a first step in the investigation of the vitality of Ukrainian in the diasporic communities.

## 6 Conclusion and future directions

Our study aimed to explore Ukrainian–Russian bilingualism within the context of current war-affected migration from Ukraine to Austria and Germany. Addressing a gap in research on Ukrainian–Russian bilingualism in diasporic communities, particularly in Austria and Germany, we examined language attitudes among migrants and refugees toward Ukrainian and Russian. The survey collected demographic information, language proficiency, attitudes, and language use data from 406 Ukrainians in two host countries (Austria: *n* = 103; Germany: *n* = 306). We compared self-rated proficiency in Ukrainian and Russian and analyzed attitudes and language use. Additionally, network modeling analysis was conducted to understand the relationships between these variables, revealing proficiency in Ukrainian and Russian as the strongest nodes affecting language use and attitudes toward the respective languages.

We focused on the granted symbolic and pragmatic values of the both languages due to their relevance for language vitality in multilingual communities. In the case of Ukrainian–Russian bilingualism transferred from the homeland to the host countries and intensively studied in relation to Ukraine, the correlation between the two values for Ukrainian and Russian, respectively, is especially relevant. Our study has identified an imbalance between the symbolic and pragmatic values of both languages through the evaluation of attitudes: While Ukrainian has gained a higher symbolic status, Russian retains a more favorable pragmatic status as a *lingua franca* in the diasporic context, despite its negative symbolic status. At the same time, we expect that the increasing symbolic value of Ukrainian and the diminishing value of Russian will lead to an increase in the use of Ukrainian also in predominantly Russian-dominant speakers of Ukrainian migrants and refugees, even as an insider-code in hermetic minority groups. However, being a societal language in both Austria and Germany, German occupies a top position in the functional ranking of languages used in the host countries, exerting significant influence on the dynamics of transferred postcolonial bilingualism. Due to German’s unparalleled pragmatic value, diasporic Ukrainian–Russian bilingualism may undergo restructuring and reduction, particularly in subsequent generations, and, in doing so, share the destiny of other migrant heritage languages. The extent and direction of this linguistic shift are subjects for further investigation, exploring which language gains prominence and which may be marginalized in this process.

Notwithstanding the fact that languages serve as the prime marker of national identity and the engine for the creation of nation-states promoting the use of one official (national) language within that state, multilingualism turns out to be more resilient than anticipated. The pragmatic usefulness of a language easily surpasses the political necessity of one language in one state. This is particularly the case in diasporic and post-colonial situations, where languages are challenged by surrounding dominant languages or the comfort of *a lingua franca* (often the (ex)colonial language). As such, Ukrainian–Russian bilingualism in diasporic communities is not different from other, similar situations comprising other cases of post-Soviet Russian-based bilingualism in Germany (Levkovych, 2015), or of other migrant and post-colonial communities (Gatrell, 2019), e.g., in Australia (Hajek and Slaughter, 2014), or in India (Sandhu and Higgins, 2016). Our study corroborates theses authors’ argument that when a national discourse meets a transnational one, the commodification of language and identities becomes one of the key mechanisms of adaptation and integration of migrant communities within the context of globalization (Heller, 2010; Park and Wee, 2013). At the same time, our study indicates that in the context of war shifts in the symbolic values of languages in migrant communities are in line with the shifts observed in the national context, i.e., in the homeland.

Furthermore, our study has demonstrated a well-established, yet worth-reiterating finding: Bilingualism as a practice of language use in general does not pose a threat to the maintenance of any language in the diaspora; the crucial factors influencing language vitality are language proficiency, institutional support, prestige, and demographics.

As the first of its kind, our study provides a first step in shedding light on the vitality and use of Ukrainian in migrant communities in the context of the war. In doing so, our study also contributes to the investigation of post-colonial bilingualism transferred from the homeland into the host country, i.e., of a special case of multilingualism, cf. e.g., Kazakh–Russian bilingualism in Germany (Zhakupova, 2014). The applied concept of the (im)balance of symbolic and pragmatic values of languages as a factor of their vitality in a multilingual environment, mainly used in colonial linguistics (De Kadt, 1991, 1993), proves to be fruitful in relation to diasporic communities. By drawing on insights from (post)colonial linguistics and research on languages and war—albeit without strictly adhering to any one specific theoretical paradigm—we want to start the debate about transferred bilingualism within diaspora communities, notably in post-Soviet, Russian-based bilingualism in migrant communities.

Unlike translation studies (Zhdanova, 2009) or studies on languages in the First World War (Declercq and Walker, 2016), migrant linguistics has paid very little attention to the issue of languages and war. Our study shows that it is crucial for understanding multilingualism and migration, especially in the context of war, when language, as key to (linguistic) nationalism, obtains a political dimension. As other cases across time and space have shown already, war always affects language attitudes which, in turn, can lead to shifts in language use.

## Data availability statement

The raw data supporting the conclusions of this article will be made available by the authors, without undue reservation.

## Ethics statement

The studies involving humans were approved by Ethikkommission (Ethics Committee), University of Potsdam. The studies were conducted in accordance with the local legislation and institutional requirements. The participants provided their written informed consent to participate in this study.

## Author contributions

VW: Writing – review & editing, Writing – original draft. NM: Writing – review & editing, Writing – original draft.

## Footnotes

^1^Cf. the survey-based linguistic-geographical mapping in Ukraine in Hentschel and Taranenko (2021)

^2^Cf. the most recent reflections on the development and measurability of the linguistic vitality in Ding (2023), Jamallullail and Nordin (2023) and Clément and Norton (2021).

^3^See the comparable issues to the pragmatic status of Russian on the territory of the former USSR in Alpatov (1997), shortly summarized, (e.g., in Pavlenko, 2013), to the role of symbolic and pragmatic values of Belarusian and Russian in the Belarusian political protests in Warditz and Goritskaya (2021), and to “the case of Dagestan” in Dobrushina and Kultepina (2021).

^4^Acquisition of German as a societal language in both host countries can consequently contribute to the reduced use of home languages. Time will show, whether Ukrainian as a L2 in Russian-dominant migrants and refugees vs. Russian as a L2 in Ukrainian-dominant migrants and refugees will be given up, and whether the growing symbolic value of Ukrainian will contribute to a language shift in Russian-dominant migrants and refugees.

^5^For the initial discussion of the (im)balance between *symbolic and pragmatic power* regarding minority language communities (see e.g., De Kadt, 1991, 1996) based, in turn, on the reflection of Bourdieu’s concept (Bourdieu, 1991).

## References

[ref010] Abacioglu C. S. Isvoranu A. M. Verkuyten M. Thijs J. Epskamp S. (2019). Exploring multicultural classroom dynamics: A network analysis. J. Sch. Psychol. 74, 90–105. 31213234 10.1016/j.jsp.2019.02.003

[ref1] Abd El Jawad H. R. (2006). Why do minority languages persist? The case of Circassian in Jordan. Int. J. Biling. Educ. Biling. 9, 51–74. doi: 10.1080/13670050608668630

[ref2] Achterberg J. (2005). Zur Vitalität slavischer Idiome in Deutschland. Eine empirische Studie zum Sprachverhalten slavophoner Immigranten. München: Otto Sagner.

[ref3] Albarracin D. Shavitt S. (2018). Attitudes and attitude change. Annu. Rev. Psychol. 69, 299–327. doi: 10.1146/annurev-psych-122216-011911, PMID: 28841390 PMC12479097

[ref4] Albrecht C. Panchenko T. (2022). “Fluchtbewegungen aus der Ukraine: Ursachen, Auswirkungen und Prognosen” in ifo Schnelldienst. München: ifo Institut. 29–36.

[ref5] Alpatov V. (1997). 150 yazykov i politika (1917–1997). Sociolingvističeskie problemy SSSR i postsovetskogo prostranstva. Moskva: Institut vostokovedeniya RAN.

[ref6] Azhniuk B. (Ed.) (2019). Movne zakonodavstvo i movna polityka: Ukraina, Yevropa, svit (Zbirnyk naukovykh prats). Kyiv: Vydavnychyi dim Dmytra Buraho.

[ref7] Besters Dilger J. (2009). Language policy and language situation in Ukraine: analysis and recommendations. Frankfurt am Main: Peter Lang.

[ref8] Bilaniuk L. (2005). Contested tongues: language politics and cultural correction in Ukraine. Ithaca and London: Cornell University Press.

[ref9] Bourdieu P. (1991). Language and symbolic power. Cambridge: Polity Press.

[ref10] Bourhis R. Y. Sachdev I. Ehala M. Giles H. (2019). Assessing 40 years of group vitality research and future directions. J. Lang. Soc. Psychol. 38, 409–422. doi: 10.1177/0261927X19868974, PMID: 30257663

[ref11] Bradley D. (2013). “Language attitudes: the key factor in language maintenance” in Language endangerment and language maintenance: an active approach. eds. Bradley D. Bradley M.. (Bradley: Routledge), 1–10.

[ref12] Brown I. Sachdev I. (2009). Bilingual behavior, attitudes, identity and vitality: some data from Japanese speakers in London, UK. J. Multiling. Multicult. Dev. 30, 327–343. doi: 10.1080/01434630902780715

[ref001] Bringmann L. F. Elmer T. Epskamp S. Krause R. W. Schoch D. Wichers M.. (2019). What do centrality measures measure in psychological networks? J. abnor. psychol. 128:892. 10.1037/abn0000446 31318245

[ref13] Budzhak Jones S. (1998). Against word-internal codeswitching: evidence from Ukrainian–English bilingualism. Int. J. Biling. 2, 161–182. doi: 10.1177/136700699800200203

[ref14] Burda T. (2002). Movna povedinka osobystosti v umovakh ukrainsko–rosiiskoho bilinhvizmu (molodizhne seredovyshche m. Kyieva). Dys. kand. filol. nauk. Kyiv: Instytut ukrainskoi movy NAN Ukrainy (manuskrypt).

[ref15] Clément R. Norton B. (2021). Ethnolinguistic vitality, identity and power: investment in SLA. J. Lang. Soc. Psychol. 40, 154–171. doi: 10.1177/0261927X20966734

[ref16] Connelly J. (2020). From peoples into nations: a history of Eastern Europe. Princeton: Princeton University Press.

[ref17] Csernicskó I. Fedinec C. (2016). Four language Laws of Ukraine. Int. J. Minor. Group Rights 23, 560–582. Available at: https://www.jstor.org/stable/26557843

[ref18] Csernicskó I. Kontra M. (2022). “The linguistic human rights plight of Hungarians in Ukraine” in The handbook of linguistic human rights. eds. Skutnabb Kangas T. Phillipson R. (Hoboken, NJ: Wiley-Blackwell), 373–382.

[ref19] De Kadt E. (1991). Language, power and emancipation: a south African perspective. Theoria 78, 1–15.

[ref20] De Kadt E. (1993). Language, power, and emancipation in South Africa. World Englishes 12, 157–168. doi: 10.1111/j.1467-971X.1993.tb00018.x

[ref21] De Kadt E. (1996). Language and apartheid: the power of minorities. Alternation 3, 184–194.

[ref22] Declercq C. Walker J. (2016). Languages and the first world war: representation and memory. London: Palgrave Macmillan.

[ref23] Del Gaudio S. (2015). Ukrainsko–russkaia smeshannaia rech “Surzhik” v sisteme vzaimodeistviia ukrainskogo i russkogo iazykov. Slověne 2, 214–241. Available at: http://slovene.ru/ojs/index.php/slovene/article/view/117

[ref24] Ding S. L. (2023). Rethinking marginalization and heritage language vitality in multilingual families. Int. J. Biling. 27, 603–617. doi: 10.1177/13670069221111861

[ref25] Dobrushina N. Kultepina O. (2021). The rise of a lingua franca: the case of Russian in Dagestan. Int. J. Biling. 25, 338–358. doi: 10.1177/1367006920959717

[ref26] Dragojevic M. Fasoli F. Cramer J. Rakić T. (2021). Toward a century of language attitudes research: looking back and moving forward. J. Lang. Soc. Psychol. 40, 60–79. doi: 10.1177/0261927X20966714

[ref27] Epskamp S. Borsboom D. Fried E. I. (2018). Estimating psychological networks and their accuracy: a tutorial paper. Behav. Res. Methods 50, 195–212. doi: 10.3758/s13428-017-0862-1, PMID: 28342071 PMC5809547

[ref002] Epskamp S. Isvoranu A. M. (2022). New trends in network modeling of psychopathology. World Psychiatry. 21:463. 36073689 10.1002/wps.21017 PMC9453883

[ref28] Forker D. Grenoble L. (Eds.) (2021). Language contact in the territory of the former Soviet Union. Amsterdam: John Benjamins.

[ref29] Fridman C. Livni A. Bar On S. Meir N. (2024). Modeling lexical abilities of heritage language and L2 speakers of Hebrew and English in Israel and the United States: a network approach. Front. Psychol. 15:1331801. doi: 10.3389/fpsyg.2024.1331801 38778883 PMC11110673

[ref30] Friedman A. D. (2023). Defending borders and crossing boundaries: ideologies of polylanguaging in interviews with bilingual Ukrainian youth. Int. J. Multiling. 20, 623–639. doi: 10.1080/14790718.2021.1874386

[ref31] Gatrell P. (2019). The unsettling of Europe: the great migration, 1945 to the present. New York: Basic Books.

[ref32] Giles H. Bourhis R. Taylor D. M. (1977). “Towards a theory of language in ethnic group relations” in Language, ethnicity and intergroup relations. ed. Giles H. (Cambridge, MA: Academic Press), 307–348.

[ref33] Hajek J. Slaughter Y. (Eds.) (2014). Challenging the monolingual mindset. Bristol: Blue Ridge Summit.

[ref34] Halko Addley A. Khanenko Friesen N. (2019). Language use and language attitude among Ukrainian Canadians on the prairies: an ethnographic analysis. East/West: J. Ukr. Stud. VI 2, 53–71. doi: 10.21226/ewjus530

[ref35] Heller M. (2010). The commodification of language. Annu. Rev. Anthropol. 39, 101–114. doi: 10.1146/annurev.anthro.012809.104951

[ref37] Hentschel G. Brüggemann M. (2015). “Gibt es in der Ukraine einen ukrainisch–russischen Sprachkonflikt? Über das Miteinander, Gegeneinander und Durcheinander von Ukrainisch und Russisch” in Ukraine. Krisen. Perspektiven. Interdisziplinäre Betrachtungen eines Landes im Umbruch. eds. Bakalova E. Endrich T. Kh S. Spodarets G. (Berlin: wvb Wissenschaftlicher Verlag), 95–118.

[ref38] Hentschel G. Taranenko O. (2021). Bilingualism or tricodalism: Ukrainian, Russian and “Suržyk” in Ukraine: analysis and linguistic-geographical mapping. Die Welt der Slaven 66, 268–299. doi: 10.13173/WS.66.2.268

[ref39] Hentschel G. Zeller J. (2016). Meinungen und Einstellungen zu Sprachen und Kodes in zentralen Regionen der Ukraine. Z. Slaw. 61, 636–661. doi: 10.1515/slaw-2016-0039

[ref40] Jamallullail S. H. Nordin S. M. (2023). Ethnolinguistics vitality theory: the last stance for a language survival. Sustain. Multiling. 22, 27–55. doi: 10.2478/sm-2023-0002

[ref41] Kircher R. (2022). “Questionnaires to elicit quantitative data” in Research methods in language attitudes. eds. Kircher R. Zipp L. (Cambridge: Cambridge University Press), 129–144.

[ref42] Kircher R. Zipp L. (Eds.) (2022). Research methods in language attitudes. Cambridge: Cambridge University Press.

[ref43] Kosmarskaya N. (2005). “Post-Soviet Russian diaspora” in Encyclopedia of diasporas. eds. Ember M. Ember C. R. Skoggard I. (Boston: Springer).

[ref44] Kulyk V. (2017). “Language attitudes in independent Ukraine: differentiation and evolution” in Harvard Ukrainian Studies (Harvard Ukrainian Research Institute), 265–292.

[ref45] Kulyk V. (2023). Die Ukrainer sprechen jetzt hauptsächlich Ukrainisch—sagen sie. Ukraine–Analysen 284, 2–5.

[ref46] Leerssen J. (2010). National thought in Europe. A cultural history. 3rd Edn. Amsterdam: Amsterdam University Press.

[ref47] Levkovych N. (2015). “On the linguistic behavior of immigrants from the post-soviet countries in Germany” in Language empires in comparative perspective. ed. Stolz C. (Berlin: De Gruyter), 285–298.

[ref48] Marusyk T. (2019). “Zdobutky i vtraty movnoi polityky v umovakh priamoi voiennoi ahresii ta hibrydnoi viiny” in Movne zakonodavstvo i movna polityka: Ukraina, Yevropa, svit (Zbirnyk naukovykh prats). ed. Azhniuk B. (Kyiv: Vydavnychyi dim Dmytra Buraho), 175–188.

[ref49] Masenko L. (2020a). Konflikt mov ta identychnostei u postradianskii Ukraini. Kyiv: Vydavnychyi dim “Klio”.

[ref50] Masenko L. (2020b). “Language conflict in Ukraine: finding of settlement” in Discourse and practice of bilingualism. Contemporary Ukraine and Russia/Tatarstan. eds. Müller D. Wingender M. (Wiesbaden: Harrassowitz), 31–42.

[ref51] Meir N. Joffe S. Shabtaev R. Walters J. Armon-Lotem S. (2021). Heritage languages in Israel: the multilingual tapestry with Hebrew threads. in The Cambridge handbook of heritage languages and linguistics, ed. by Montrul S., and Polinsky M., 129–155, Cambridge: Cambridge University Press

[ref52] Miller A. I. (2006). Imperija Romanovych i nacionalizm. Ėsse po metodologii istoričeskogo issledovanija. Moskva: Novoe literaturnoe obozrenie.

[ref53] Moser M. (2013a). Language policy and the discourse on languages in Ukraine under president Viktor Yanukovych (25 February 2010–28 October 2012). Stuttgart: ibidem Press.

[ref54] Moser M. (2013b). “The “Mirror from Overseas”: the history of modern standard Ukrainian as reflected in the North American Ukrainian Newspaper Svoboda (the early years: from 1893 to the 1930s)” in Slavic languages in migration. eds. Moser M. Polinsky M. (Wien: LIT Verlag), 43–80.

[ref55] Moser M. (2015). “Pushing the “regional language”–Ukraine’s law “on principles of the state language policy” in force” in Ukraine twenty years after independence: assessments, perspectives, challenges. eds. Brogi G. E. Dyczok M. Pachlovska O. Siedina G. (Aracne editrice: Roma), 189–211.

[ref56] Mykhaylyk R. Ytterstad E. (2017). Directionality of cross-linguistic influence: which referring choices do bilingual Ukrainian–English children make? Int. J. Biling. 21, 99–121. doi: 10.1177/1367006915603824

[ref57] Nagy N. (2018). Linguistic attitudes and contact effects in Toronto’s heritage languages: a variationist sociolinguistic investigation. Int. J. Biling. 22, 429–446. doi: 10.1177/1367006918762160

[ref58] Neroznak V. (Ed.) (1994). Krasnaja kniga jazykov narodov Rossii: ėnciklopedičeskij slovar’ spravočnik. Moskva: Academia.

[ref59] Neroznak V. (Ed.) (1995). Gosudarstvennye jazyki v Rossijskoj Federacii: ėnciklopedičeskij slovar’–spravočnik. Moskva: Academia.

[ref60] Olszański T. (2012). The language issue in Ukraine. An attempt at a new perspective. Warsaw: OSW Studies.

[ref61] Panagiotidis J. (2020). Postsowjetische Migration in Deutschland: Eine Einführung. Weinheim: Beltz Juventa Verlag.

[ref62] Park J. S. Wee L. (2013). Markets of English: linguistic capital and language policy in a globalizing world. London: Routledge.

[ref63] Pavlenko A. (2013). Multilingualism in post-soviet successor states. Lang. Linguist. Compass 7, 262–271. doi: 10.1111/lnc3.12024

[ref64] Racek D. Davidson B. I. Thurner P. W. Zhu X. X. Kauermann G. (2024). The Russian war in Ukraine increased Ukrainian language use on social media. Commun. Psychol. 2:1. doi: 10.1038/s44271-023-00045-6 PMC11332000 39242855

[ref65] Rahman T. (2001). Language-learning and power: a theoretical approach. Int. J. Sociol. Lang. 152, 53–74. doi: 10.1515/ijsl.2001.057

[ref66] Sandhu P. Higgins C. (2016). “Identity in post-colonial contexts” in The Routledge handbook of language and identity. ed. Preece S. (London: Routledge), 179–194.

[ref67] Scheer T. (2022). Die Sprachenvielfalt in der österreichisch–ungarischen Armee (1867–1918). Wien: Heeresgeschichtliches Museum.

[ref68] Shang G. (2020). Wrestling between English and pinyin: language politics and ideologies of coding street names in China. J. Lang. Politics 19, 624–645. doi: 10.1075/jlp.19072.sha

[ref69] Shevchenko N. (2014). L’histoire du bilinguisme en Ukraine et son rôle dans la crise politique d’aujourd’hui. Cahiers Sens 17–18, 203–225. doi: 10.3917/csp.017.0203

[ref70] Shevelov G. (1966). Die Ukrainische Schriftsprache 1798–1965. Ihre Entwicklung unter dem Einfluss der Dialekte. Wiesbaden: Otto Harrassowitz.

[ref71] Sokolova S. (2019). “Ukrainska mova v ofitsiinii sferi: problemy i perspektyvy” in Movne zakonodavstvo i movna polityka: Ukraina, Yevropa, svit (Zbirnyk naukovykh prats). ed. Azhniuk B. (Vydavnychyi dim Dmytra Buraho: Kyiv), 160–174.

[ref72] Sokolova S. O. Zalizniak H. M. (2018). Osoblyvosti suchasnoi movnoi sytuatsii Ukrainy u dzerkali sotsiolohii ta sotsiolinhvistyky. Ukrainska mova 3, 3–19. doi: 10.15407/ukrmova2018.02

[ref73] Stolz C. (Ed.) (2015). Language empires in comparative perspective. Berlin: De Gruyter.

[ref003] Taranenko O. (2001). Ukrainska mova i suchasna movna sytuatsiia v Ukraini. Movoznavstvo 4, 3–19.

[ref74] Thomason S. (2008). Social and linguistic factors as predictors of contact-induced change. J. Lang. Contact 2, 42–56. doi: 10.1163/000000008792525381

[ref75] Thomason S. G. (2017). “Contact as a source of language change” in The handbook of historical linguistics. eds. Brain D. J. Janda R. D. (Oxford: Blackwell), 687–712.

[ref76] Thomason S. G. Kaufman T. (1988). Language contact, creolization, and genetic linguistics. Berkeley: University of California Press.

[ref004] Tkachenko O. (1999). Chy mozhut buty v Ukraini dvi zahalnoderzhavni movy? Movoznavstvo 4–5, 3–9.

[ref77] Trub V. (2000). Javišče “suržyku” jak forma prostoričč’a v situaciji dvomovnosti. Movoznavstvo 1, 46–58.

[ref78] Walker J. Declercq Ch. (eds.) (2016). Languages and the First World War: Communicating in a Transnational War. London: Palgrave Macmillan.

[ref79] Warditz V. (2013). “Jazyki slavjanskich diaspor: aktual’nyj status, problemy i perspektivy izučenija” in Deutsche Beiträge zum 15. Internationalen Slavistenkongress, Minsk 2013. eds. Kempgen S. Wingender M. Franz N. Jakiša M. (München-Berlin-Washington: Otto Sagner), 303–312.

[ref80] Warditz V. (2020). “Slavic languages in diaspora” in Encyclopedia of Slavic languages and linguistics online. eds. Greenberg M. L. Grenoble L. A. (Brill Encyclopedia). Available at: https://referenceworks.brill.com/display/entries/ESLO/COM-036122.xml

[ref81] Warditz V. Goritskaya O. (2021). “Sociolingvistika v protestnom dviženii Belarusi” in Kommunikacija v ėpochu protestov. eds. Norman B. Kuße H. (Berlin: Peter Lang), 25–60.

[ref82] Zalbidea J. Cabo D. P. Loza S. Luque A. (2023). Spanish heritage language learners’ motivational profile in the postsecondary classroom: insights from psychological network modeling. Stud. Second. Lang. Acquis. 45, 979–1003.

[ref83] Zhabotynska S. A. (2018). Dominantnist ukrainskoi movy v umovakh biliinhvizmu: Neirokohnityvni chynnyky. Visnyk Kharkivskoho Natsionalnoho Universytetu imeni V. N. Karazina: Inozemna Filolohiia. Metodyka Vykladannia Inozemnykh Mov 87, 5–19. doi: 10.26565/2227-8877-2018-87-01

[ref84] Zhakupova A. D. (Ed.) (2014). Jazyk, kul’tura, diaspora: kazachi Evropy. Kokštenau: Izdatel’stvo KGU im. Š. Ualichanova.

[ref85] Zhdanova V. (2009). “Unsere Waffe war das Wort…” Translation in Kriegszeiten. Frankfurt am Main: Peter Lang.

