# Supplementary material for: Ukrainian–Russian bilingualism in the war-affected migrant and refugee communities in Austria and Germany: a survey-based study on language attitudes
Source: Front Psychol. 2024 May 23;15:1364112. doi: 10.3389/fpsyg.2024.1364112 (PMC11154112; doi:10.3389/fpsyg.2024.1364112)
Supplement: Supplementary file 1 [file Data_Sheet_1.docx]

**Appendix**

**Appendix 1. Questionnaire in Russian**

**Анкета**

1. **Общие сведения**

Ваш возраст: ________________________

Ваш пол:  мужской  женский  предпочитаю не указывать

В каком городе вы живете сейчас? _______________________

В каком году вы переехали в Германию/ Австрию? _______________________

В каком городе/ селе Украины вы жили до переезда в Германию/ Австрию?_____________________

Ваш уровень образования:

□ Базовое общее среднее образование (9 классов)

□ Полное среднее образование (11 классов)

□ Профессионально–техническое образование (колледж или техникум)

□ Высшее образование – бакалаврский уровень

□ Высшее образование – магистерский уровень

□ Высшее образование – доктор философии

□ Другое __________________________

Кем вы работаете? Если в настоящее время Вы не работаете, укажите, пожалуйста, последнее место работы_____________________________________

1. **Информация о владении языками**

Ваш родной(–ые) язык(–и)? ________________________________________

Вы росли в двуязычной семье? Если да, на каких языках говорили с вами в семье?

□ Нет

□ Да: ____________________________________

Насколько хорошо вы владеете указанными ниже языками?

*Для каждого языка отметьте одну цифру на шкале от 1 до 10, где 1 – начальный уровень владения языком, а 10 – уровень носителя. Если вы с детства говорили еще на одном или нескольких языках или учили их в школе, также заполните таблицу для этих языков.*

| **Владение языком** | **1** | **2** | **3** | **4** | **5** | **6** | **7** | **8** | **9** | **10** |
| --- | --- | --- | --- | --- | --- | --- | --- | --- | --- | --- |
| Украинский | □ | □ | □ | □ | □ | □ | □ | □ | □ | □ |
| Русский | □ | □ | □ | □ | □ | □ | □ | □ | □ | □ |
| Немецкий | □ | □ | □ | □ | □ | □ | □ | □ | □ | □ |
| Другой язык:______________________ | □ | □ | □ | □ | □ | □ | □ | □ | □ | □ |
| Другой язык:______________________ | □ | □ | □ | □ | □ | □ | □ | □ | □ | □ |

1. **Языковые установки**

Насколько вы согласны или не согласны со следующими заявлениями?

*Для каждого утверждения и языка выберите цифру по шкале от 1 до 5, которая наиболее отвечает вашей позиции:*

*1 – совершенно не согласен(–сна)*

*2 – не согласен(–сна)*

*3 – затрудняюсь ответить*

*4 – согласен(–сна)*

*5 – полностью согласен(–сна)*

*В начале таблицы приведен пример заполнения. В некоторых случаях, возможно, ваше мнение в отношении обоих языков будет совпадать. В таком случае просто поставьте одинаковые цифры в колонки с обоими языками.*

|  | Язык 1 | Язык 2 |
| --- | --- | --- |
| Пример:  *Я говорю на этом языке с рождения.* | 1 | 5 |
|  | **Украинский** | **Русский** |
| Знание этого языка – важная часть моей идентичности. |  |  |
| Мне важно, чтобы мои дети знали этот язык. |  |  |
| Я предпочитаю говорить на этом языке. |  |  |
| В Германии/ Австрии мало возможностей для поддержки знаний этого языка. |  |  |
| В Германии/ Австрии я чаще использую этот язык. |  |  |
| Этот язык вызывает во мне положительные эмоции. |  |  |
| Я чувствую, что могу выразить свои эмоции во всей полноте на этом языке. |  |  |
| Я не чувствую себя самим собой, когда говорю на этом языке. |  |  |
| Иногда я чувствую, что мне не хватает словарного запаса, когда я говорю на этом языке. |  |  |
| Война и политические события последних лет изменили мое отношение к этому языку в худшую сторону. |  |  |
| Я хотел(–а) бы полностью отказаться от использования этого языка. |  |  |

**Appendix 2. Questionnaire in Ukrainian**

**Анкета**

1. **Загальні відомості**

Ваш вік: ________________________

Ваша стать:  чоловіча  жіноча  не хочу вказувати

У якому місті ви мешкаєте зараз?_______________________

Коли ви приїхали до Німеччини/ до Австрії? Вкажіть точну дату_______________________

У якому місті чи селі України Ви жили до переїзду у Німеччину/ в Австрію?_____________________

1. Вкажіть Ваш рівень освіти:

□ Базова загальна середня освіта (9 клас)

□ Повна загальна середня освіта (11 клас)

□ Професійно–технічне навчання (коледж)

□ Вища освіта – бакалавр

□ Вища освіта – магістр

□ Вища освiта – доктор філософії

□ Iнше ________________________

Ким Ви працюєте? Якщо Ви не працюєте, вкажіть, будь ласка, останнє місце роботи _____________________________________

1. **Інформація про знання мови**

Яка(i) Ваша(–i) рідна(–i) мова(–и)? ________________________________________________

1. Ви росли у двомовній родинi? Якщо так, якими мовами розмовляли з Вами у вашій родинi?

□ Нi

□ Так: ____________________________________

1. Наскільки добре Ви говорите наступними мовами? Для кожної мови позначте одну цифру на шкалi від 1 до 10, де 1 – початковий рівень володіння мовою, а 10 – рівень рідної мови. Якщо Ви говорили або вчили одну або декілька мов з дитинства, будь ласка, заповніть таблицю для цих мов.

| **Володіння мовою** | **1** | **2** | **3** | **4** | **5** | **6** | **7** | **8** | **9** | **10** |
| --- | --- | --- | --- | --- | --- | --- | --- | --- | --- | --- |
| Українська | □ | □ | □ | □ | □ | □ | □ | □ | □ | □ |
| Російська | □ | □ | □ | □ | □ | □ | □ | □ | □ | □ |
| Німецька | □ | □ | □ | □ | □ | □ | □ | □ | □ | □ |
| Інша мова:_____________________ | □ | □ | □ | □ | □ | □ | □ | □ | □ | □ |
| Інша мова:____________________ | □ | □ | □ | □ | □ | □ | □ | □ | □ | □ |

1. **Мовні настанови**

Наскільки Ви згодні чи не згодні з наступними заявами?

Для кожного твердження та мови виберіть цифру за шкалою від 1 до 5, яка найбільше відповідає Вашій позиції:

*1 – зовсім не згоден (не згодна)*

*2 – не згоден (не згодна)*

*3 – важко відповісти*

*4 – згоден (згодна)*

*5 – повністю згоден (згодна)*

*На початку таблиці наведено приклад заповнення. У деяких випадках, можливо, Ваша думка щодо обох мов співпадатиме. У такому разі просто поставте однакові цифри в колонки з обома мовами.*

|  | Мова 1 | Мова 2 |
| --- | --- | --- |
| Приклад:  *З самого народження я розмовляю цією мовою.* | 1 | 5 |
|  | **Українська** | **Російська** |
| Знання цієї мови є важливою частиною моєї ідентичності. |  |  |
| Мені важливо, щоб мої діти знали цю мову. |  |  |
| Мені більше подобається говорити цією мовою. |  |  |
| У Німеччині /в Австрії мало можливостей підтримати знання цієї мови. |  |  |
| У Німеччині /в Австрії я частіше використовую цю мову. |  |  |
| Ця мова викликає у мені позитивні емоції. |  |  |
| Я відчуваю, що можу висловити свої емоції у всій повноті цією мовою. |  |  |
| Я не почуваюся самим собою, коли розмовляю цією мовою. |  |  |
| Іноді я відчуваю, що мені не вистачає словникового запасу, коли я говорю цією мовою. |  |  |
| Війна та політичні події останніх років змінили моє ставлення до цієї мови на гірше. |  |  |
| Я хотів (хотіла) би повністю відмовитись від використання цієї мови. |  |  |
